# A haplotype-resolved, chromosome-scale genome assembly and annotation for *Carya glabra* (pignut hickory; Juglandaceae)

**DOI:** 10.1093/g3journal/jkag135

**Published:** 2026-05-20

**Authors:** Shengchen Shan, Edgardo M Ortiz, Baylee Klein, Arthur Oganisyan, Gia Serrano, Bryanna Stults, Rubina Torkzadeh, Audrey Tucker, Ezra Linnan, Benjamin Pringle, Tyler Radtke, Moumita Hoque Rainy, Lia Swanson, Gabrielle Vines, Lauren Whitt, Huiting Zhang, Alex Harkess, Pamela S Soltis, Douglas E Soltis

**Affiliations:** Florida Museum of Natural History, University of Florida, Gainesville, FL 32611, United States; Florida Museum of Natural History, University of Florida, Gainesville, FL 32611, United States; Department of Computer & Information Science & Engineering, University of Florida, Gainesville, FL 32611, United States; Department of Chemistry, University of Florida, Gainesville, FL 32611, United States; Department of Chemistry, University of Florida, Gainesville, FL 32611, United States; Department of Mechanical and Aerospace Engineering, University of Florida, Gainesville, FL 32611, United States; Department of Biomedical Engineering, University of Florida, Gainesville, FL 32611, United States; Department of Biology, University of Florida, Gainesville, FL 32611, United States; Department of Statistics, University of Florida, Gainesville, FL 32611, United States; Department of Computer & Information Science & Engineering, University of Florida, Gainesville, FL 32611, United States; Department of Biology, University of Florida, Gainesville, FL 32611, United States; Department of Botany, University of Dhaka, Dhaka 1000, Bangladesh; Department of English, University of Florida, Gainesville, FL 32611, United States; Department of Entomology and Nematology, University of Florida, Gainesville, FL 32611, United States; HudsonAlpha Institute for Biotechnology, Huntsville, AL 35806, United States; Department of Horticulture, Washington State University, Pullman, WA 99164, United States; HudsonAlpha Institute for Biotechnology, Huntsville, AL 35806, United States; Florida Museum of Natural History, University of Florida, Gainesville, FL 32611, United States; Genetics Institute, University of Florida, Gainesville, FL 32610, United States; Biodiversity Institute, University of Florida, Gainesville, FL 32611, United States; Florida Museum of Natural History, University of Florida, Gainesville, FL 32611, United States; Department of Biology, University of Florida, Gainesville, FL 32611, United States; Genetics Institute, University of Florida, Gainesville, FL 32610, United States; Biodiversity Institute, University of Florida, Gainesville, FL 32611, United States

**Keywords:** autopolyploid, campus genome initiative, chloroplast genome, chromosome-level genome, comparative genomics, conservation, *Carya glabra*, haplotype-resolved, mitochondrial genome, nuclear phylogeny, undergraduate training, genome assembly

## Abstract

*Carya glabra* (2*n* = 4*x* = 64), also known as pignut hickory, is a widely distributed species in the walnut family (Juglandaceae). Native to the central and eastern United States and southeastern Canada, *C. glabra* plays an important ecological role as a common upland forest species; it is closely related to several economically valuable nut trees, including *Carya illinoinensis* (pecan). A deeper understanding of the genetics of *C. glabra* is essential for studying its evolutionary history and biology, with potential implications for agricultural improvement of pecan. Here, we present the first nuclear genome assembly and annotation of *C. glabra*. The assembly is chromosome level and phased, representing the first assembled polyploid genome in the genus *Carya*. A total of 64 pseudochromosomes were assembled and phased into 4 haplotypes. The haplotype A assembly spans 600.4 Mb, comprises 55.0% repetitive sequences, and contains 30,947 protein-coding genes, with a Benchmarking Universal Single-Copy Orthologs (BUSCO) completeness score of 97.7%. Functional annotation assigned 94.3% of haplotype A genes to gene families, and 79.7% and 86.3% of genes were annotated with Gene Ontology terms and protein domains, respectively; 635 putative plant disease resistance genes were found in haplotype A. The other 3 haplotypes exhibited similarly high-quality annotation metrics. Our genomic analyses also suggest that *C. glabra* is an autotetraploid. Comparative genomic analyses revealed high collinearity among the 4 haplotypes of *C. glabra* and the published genomes of 3 other *Carya* species, although structural variation among the genomes of these species was identified. In addition, we provide an improved chloroplast genome assembly and the first mitochondrial genome for *C. glabra*. We also provide a phylogenomic tree for *Carya* based on numerous nuclear genes, showing that *C. glabra* forms a clade with other North American tetraploid species. Importantly, most members of the research team are undergraduate students; the sequenced individual is located in McCarty Woods, a Conservation Area on the University of Florida campus. This work highlights the value of genome assembly efforts as powerful tools for teaching genomics and supporting conservation initiatives. This first high-quality reference genome for *C. glabra* provides a valuable resource for studying *Carya*, a genus of significant ecological and economic importance.

## Introduction

**Fig. 1.**
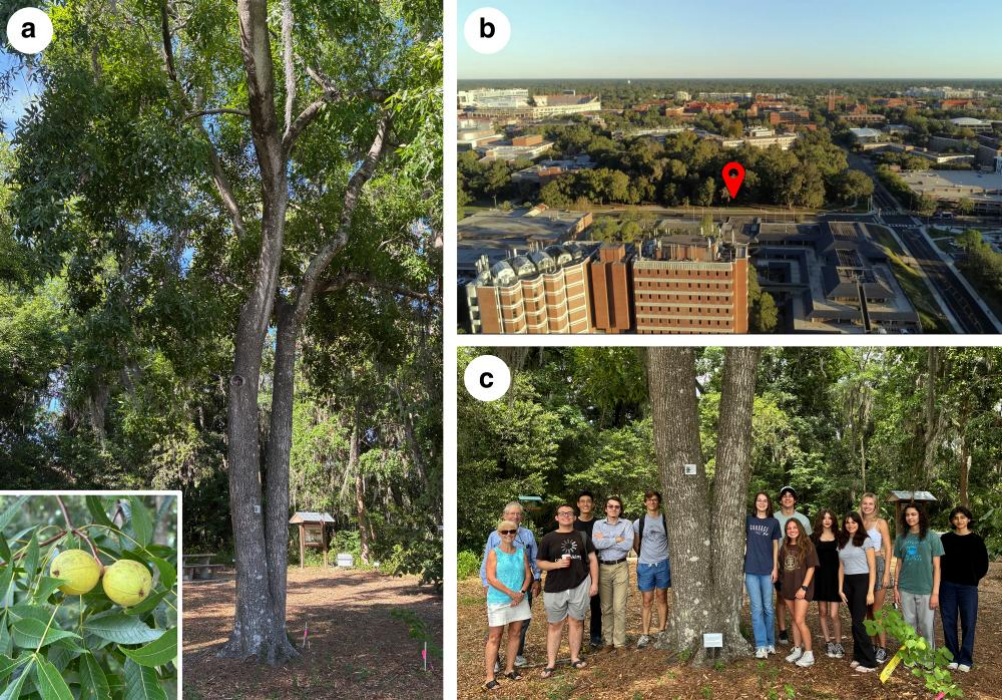
*C. glabra* (pignut hickory) on the University of Florida campus. a) The *C. glabra* individual sequenced in this study; the inset highlights the fruits and compound leaves. b) Location of the *C. glabra* individual (indicated by the red pin) in McCarty Woods on the University of Florida campus. c) Most members of the research team in front of the *C. glabra* tree; nearly all are undergraduate researchers. Photo credits: a) Shengchen Shan; b) John Rouse; c) Erin L. Grady.

*Carya glabra* (Miller) Sweet (2*n* = 4*x* = 64) (Juglandaceae; walnut family), commonly known as pignut hickory, is a widespread species in the central and eastern United States and southeastern Canada, ranging from Ontario southward to central Florida (Fig. 1a; POWO 2025). Pignut hickory is a slow-growing, deciduous tree that typically reaches 20 to 30 m in height and 30 to 100 cm in diameter (Tirmenstein 1991). The species is monoecious, bearing staminate catkins and pistillate flowers that appear in spikes (Tirmenstein 1991). *Carya* possesses an accessory fruit; a pear-shaped nut is enclosed in a 4-valved husk (of bracts). The fruit remains green until maturity, turning brown as it ripens (Fig. 1a; Smalley 1990).

The species is ecologically dominant in dry upland forests (Smalley 1990). In addition, the nuts are rich in crude fat and are consumed by a variety of wildlife, including squirrels, birds, foxes, rabbits, and raccoons (Smalley 1990). The wood of *C. glabra* is heavy and strong, making it ideal for tool handles and mallets, and it is also commonly used as fuelwood (Smalley 1990; Tirmenstein 1991). Pignut hickory also shows potential value for restoration of disturbed sites, as it has been reported to recolonize abandoned strip mines (Hardt and Forman 1989).

*Carya* comprises 19 species with an intercontinentally disjunct distribution (POWO 2025). In Asia, the genus is native to India, China, and countries in Southeast Asia, while in North America, it occurs in eastern Canada, central and eastern United States, and Mexico (POWO 2025). Phylogenetic analyses support 2 monophyletic groups within the genus, corresponding to the primary geographic distributions (Asia and North America) (Zhang et al. 2013, 2024b; Xi et al. 2022). According to molecular age estimation and biogeographic analyses, *Carya* in North America dates to the early Paleocene (Zhang et al. 2013). Its earliest confirmed occurrence is evidenced by fossil fruits from the late Eocene (Manchester 1999). The highest species diversification rate of the North America clade occurred around 10.1 million years ago (Ma) during the late Miocene, suggesting that *C. glabra* or its ancestor likely emerged around this time (Zhang et al. 2013). At least 6 North American *Carya* species, including *C. glabra*, are tetraploid (2*n* = 4*x* = 64) (Woodworth 1930; Stone 1961; Zhang et al 2013), whereas all Asian species investigated are diploid (Grauke et al. 2016). The North American clade showed a higher diversification rate than the Asian clade, which may be attributed to the polyploid nature of many North American species (Zhang et al. 2013).

Recent phylogenetic studies indicate that the closest relative of *C. glabra* may be *Carya texana*, which is also a tetraploid (Huang et al. 2019; Xi et al. 2022). Based on plastome data, other close relatives include diploid *Carya palmeri* (2*n* = 2*x* = 32) and some but not all populations of *Carya illinoinensis* (2*n* = 2*x* = 32) (Xi et al. 2022). In contrast, phylogenetic analyses, using approximately 10× resequencing data relative to the *Carya cathayensis* genome, indicate that the clade containing *C. glabra* and *C. texana* is sister to another tetraploid species, *Carya tomentosa* (Huang et al. 2019). Notable reported examples of natural hybridization involving *C. glabra* include the hybrid *Carya × demareei* Palmer, which arose from a cross between *C. glabra* and diploid *Carya cordiformis* (Sutton and Crowley 2020). Furthermore, the overlapping geographical ranges of *C. glabra* and tetraploid *Carya ovalis* have led to frequent hybridization between those 2 species (Coder 2023).

*Carya* includes 2 species that are commercially cultivated nut trees: *C. illinoinensis* (pecan) and *C. cathayensis* (Chinese hickory) (Grauke et al. 2016). In the United States, pecan production exceeded 120,000 metric tons in 2024, with a value of $468 million (USDA-NASS 2025). To date, genome assemblies have been reported for 4 *Carya* species—*C. illinoinensis* (Huang et al. 2019; Lovell et al. 2021; Xiao et al. 2021), *C. cathayensis* (Huang et al. 2019; Zhang et al. 2024b), *Carya sinensis* (Zhang et al. 2024b), and *Carya hunanensis* (Zhang et al. 2026)—all of which are diploid.

In this study, we assembled and annotated the first nuclear genome of tetraploid *C. glabra*. This chromosome-level, phased genome represents the first polyploid genome reported within the genus. The reference genome of *C. glabra* should enable novel research in the economically important genus *Carya*, with broad applications in both agriculture and evolutionary biology. The sequenced individual is located in McCarty Woods, a designated Conservation Area and quiet oasis at the center of the University of Florida (UF) campus (Fig. 1b). Most of the researchers involved in this project are undergraduate students enrolled in a Course-based Undergraduate Research Experience (CURE) class at UF (Fig. 1c). As part of the American Campus Tree Genomes (ACTG) project (https://www.hudsonalpha.org/actg), this work highlights the potential of genome assembly projects to support conservation efforts and enhance hands-on genomics education.

## Materials and methods

### Sample collection

Fresh leaf and axillary bud tissues were collected from a single *C. glabra* individual in the McCarty Woods Conservation Area, located centrally on the UF campus. The leaf tissue for DNA isolation was collected on April 5, 2024, and leaf and axillary bud tissues for RNA isolation were collected on September 10, 2025. An herbarium voucher for this plant was deposited in the Florida Museum of Natural History Herbarium (FLAS; collection number 3165, Soltis D. and Shan). The collected tissues were immediately frozen in liquid nitrogen.

### DNA isolation and sequencing

*C. glabra* leaf tissue was sent to the HudsonAlpha Institute for Biotechnology (Huntsville, AL, USA) for DNA isolation and subsequent sequencing. High-molecular-weight DNA was extracted using the Nanobind Plant Nuclei Big DNA Kit (Circulomics-PacBio, Menlo Park, CA, USA). Isolated DNA was sheared with Megaruptor (Diagenode, Denville, NJ, USA), and fragments with a size of approximately 25 kb were selected using BluePippin (Sage Science, Beverly, MA, USA). Size-selected DNA was used to construct the PacBio sequencing library using the SMRTbell Express Template Prep Kit 2.0 (PacBio, Menlo Park, CA, USA). The library was then sequenced on 2 SMRT cells on a PacBio Revio system at HudsonAlpha to generate High-Fidelity (HiFi) reads.

In addition, an Omni-C library was constructed using flash-frozen leaf material following the Dovetail Genomics protocol (Dovetail Genomics, Scotts Valley, CA, USA). The library was sequenced on 1 S4 flow cell of the Illumina NovaSeq 6000 system (Illumina, San Diego, CA, USA) at HudsonAlpha to generate paired-end 150-bp reads. Basic statistics of PacBio HiFi data and Omni-C data were assessed using SeqKit2 (v.2.4.0; Shen et al. 2024).

### RNA isolation and sequencing

Leaf and axillary bud tissues from the same *C. glabra* individual used for DNA isolation were collected and flash frozen in liquid nitrogen. RNA was extracted from each tissue (leaf and axillary bud) using a modified CTAB method (Jordon-Thaden et al. 2015). RNA quality was assessed using a Bioanalyzer at the Interdisciplinary Center for Biotechnology Research (ICBR), UF (Gainesville, FL, USA). Two strand-specific (i.e. directional) RNA-seq libraries were prepared, and the libraries were sequenced on the Illumina NovaSeq X platform to generate paired-end 151-bp reads at ICBR. The statistics of the RNA-seq data were calculated using SeqKit2, and the raw reads were filtered using fastp (v.0.23.4; Chen et al. 2018) with default parameters.

### Chloroplast and mitochondrial genome assembly and annotation

Both organellar genomes were simultaneously assembled from PacBio HiFi reads using Oatk (v.1.0; Zhou et al. 2025). Oatk's plastome assembly graph was simplified and circularized using Bandage (v.0.8.1; Wick et al. 2015), and the resulting assembly was annotated using the web application GeSeq (https://chlorobox.mpimp-golm.mpg.de/geseq.html; Tillich et al. 2017).

The plastome annotation was further curated by comparing GeSeq's annotation with the well-annotated *Nicotiana tabacum* chloroplast genome (NCBI accession number: NC_001879), as well as 3 published *C. glabra* chloroplast genomes (BK061156; OR099205; NC_067504) (Luo et al. 2021; Xi et al. 2022; Liu et al. 2025). The chloroplast genomes were first aligned using MAFFT (v.7.490) with default parameters in Geneious Prime (2025.2.2; https://www.geneious.com). The annotation was then manually inspected and curated. For protein-coding genes, we examined reading frames, exon boundaries, and start and stop codons. Ambiguous transfer RNA (tRNA) annotations were further validated using BLAST searches in the PlantRNA 2.0 database (http://plantrna.ibmp.cnrs.fr/; Cognat et al. 2022).

Oatk's mitochondrial assembly graph could not be resolved into a single circular chromosome without excluding graph segments. Therefore, 2 circular contigs were inferred from the graph and saved as separate chromosomes using Bandage (v.0.8.1; Wick et al. 2015). These 2 mitochondrial chromosomes were annotated with the web application PMGA (http://47.96.249.172:16084/annotate.html; Li et al. 2025) using the 3 databases available in the program. Additionally, we searched plastome and mitochondrial proteins using Captus (v.1.6.1; Ortiz et al. 2023). The 4 annotation tracks, 1 from Captus and 3 from PMGA (each corresponding to 1 of the 3 databases from PMGA), were checked against each other for consistency, retaining only the best annotation (i.e. that includes start and stop codons whenever possible, longest and/or most frequently observed) in case of discrepancies.

Following manual curation, the edited GenBank files were exported from Geneious Prime and then uploaded to OGDRAW (v.1.3.1; Greiner et al. 2019) to generate the final chloroplast and mitochondrial genome annotation maps using the default parameters (except checking the “tidy up annotation” box).

### Nuclear genome profiling

Jellyfish (v.2.3.0; Marçais and Kingsford 2011) was used to count *k*-mers and generate a *k*-mer histogram (*k*-mer size: 21) from the HiFi reads. The *k*-mer histogram was then imported to GenomeScope 2.0 (http://genomescope.org/genomescope2.0/; Ranallo-Benavidez et al. 2020) to infer nuclear genome characteristics, including monoploid genome size and heterozygosity, with default parameters except setting ploidal level as 4.

### Nuclear genome assembly

Hifiasm (v.0.19.9; Cheng et al. 2021) was used to perform de novo assembly with default parameters. Both HiFi reads and Omni-C reads were used as input data. Given the polyploid nature of the *C. glabra* genome, the unitig assembly from hifiasm, which contained the genomic information from all 4 haplotypes, was used for downstream analyses.

To scaffold the unitigs, first, bwa-mem2 (v.2.2.1; Vasimuddin et al. 2019) was used to align the Omni-C reads to the unitig assembly. The resulting alignments were then analyzed with the hic_qc pipeline from Phase Genomics (Seattle, WA, USA) to assess the overall quality of the Omni-C library. Then, YaHS (v.1.1; Zhou et al. 2023) was used to perform the scaffolding process with default parameters.

Next, using the Hi-C alignment file as input, the “juicer pre” tool from YaHS and Juicer (v.1.22.01; Durand et al. 2016) were used to generate the Hi-C contact map. We then manually curated the assembly by examining the Hi-C contact map using Juicebox Assembly Tools (v.1.11.08; Dudchenko et al. 2018). Misjoin and inversion errors were manually corrected, and the orientation of chromosomes was also curated to match the published *C. illinoinensis* genome (Lovell et al. 2021). After all edits, the final genome assembly was generated using the “juicer post” tool from YaHS.

A dot plot was generated using the web application D-GENIES (https://dgenies.toulouse.inra.fr/; Cabanettes and Klopp 2018) to compare the *C. illinoinensis* genome with the assembled *C. glabra* genome. To assign scaffolds to chromosomes, the *C. glabra* scaffolds were renamed according to their alignment with the *C. illinoinensis* chromosomes. The 4 copies of each chromosome in *C. glabra* were labeled A, B, C, and D in descending order of length. Each set of 16 chromosomes with the same label (e.g. Chr01A, Chr02A, …, Chr16A) was grouped and referred to as a haplotype (e.g. haplotype A). The 64 chromosomes were therefore assigned to 4 haplotypes (A, B, C, and D). It is important to note that this haplotype assignment is artificial and does not necessarily reflect a biological haplotype, since each haplotype set may represent a mixture of chromosomes originating from different gametes. For each haplotype set, genome completeness was estimated using Benchmarking Universal Single-Copy Orthologs (BUSCO, v.5.3.0) with the eudicots_odb10 database (Manni et al. 2021).

### Nuclear genome annotation

To annotate repeat sequences, for each haplotype of the chromosome-level genome assembly, EDTA (v.2.1.0; Ou et al. 2019) was used for de novo transposable element (TE) annotation. Using the TE library generated by EDTA, RepeatMasker (v.4.1.7; Smit et al. 2013–2015) was used to identify additional repeat elements and to softmask the genome (with repeat elements written in lowercase).

For gene annotation, BRAKER3 (v.3.0.8; Gabriel et al. 2024) was used to predict protein-coding genes using the RNA-seq data from the leaf and axillary bud tissues from *C. glabra* and protein evidence from model species (Supplementary Table 1). Various BRAKER3 parameter settings were tested using the haplotype A genome (Supplementary Table 2). The setting that resulted in the highest BUSCO score (using the eudicots_odb10 database) was applied to annotate all other haplotypes (i.e. B, C, and D). In addition, for each setting, the ratio of monoexonic (single-exon) to multiexonic (multiple-exon) genes was calculated using gFACs (v.1.1.2; Caballero and Wegrzyn 2019) (Supplementary Table 2). After the initial annotation, gene models meeting any of the following criteria were filtered out using AGAT (v.1.4.2; Dainat 2022): (i) presence of a premature stop codon or (ii) absence of a start and/or stop codon. Two conventional open reading frame (ORF) length thresholds were then applied (Su et al. 2013), resulting in 2 gene sets: The first set included gene models with a minimum ORF length of 101 amino acids, and the second included gene models with a minimum ORF length of 51 amino acids. The genes were named in accordance with the guidelines proposed by Cannon et al. (2025).

Functional annotation was performed using the web application TRAPID 2.0 (Bucchini et al. 2021), with the PLAZA 4.5 dicots database (Van Bel et al. 2018) as the reference and the rosids clade selected for the similarity search. All parameters were set to default, except that “input sequences are CDS” was selected.

Lastly, Circos (v.0.69-9; Krzywinski et al. 2009) was used to visualize the genome and the associated genetic features, including gene and TE densities along the chromosomes.

### Comparative genomic analyses

Genome-level synteny analysis was performed using GENESPACE (v.1.3.1; Lovell et al. 2022) to compare the 4 *C. glabra* haplotypes with chromosome-level genome assemblies from 3 diploid *Carya* species: *C. cathayensis* (Zhang et al. 2024b), *C. illinoinensis* (Lovell et al. 2021), and *C. sinensis* (Zhang et al. 2024b). The BUSCO scores for these 3 published genomes were calculated using the eudicots_odb10 database, the same database used to assess the BUSCO score for *C. glabra*.

To identify genes shared between *C. glabra* and each of the other *Carya* species with a published genome (i.e. *C. cathayensis*, *C. illinoinensis*, and *C. sinensis*), OrthoFinder (v.2.5.5; Emms and Kelly 2019) was used. A gene from a focal *Carya* species was considered shared with *C. glabra* if it belonged to an orthogroup—defined as a group of genes descended from a single gene in the last common ancestor of the focal *Carya* species and *C. glabra*—that contained 1 or more *C. glabra* genes.

### Identification of putative disease resistance genes

Because disease resistance is a key trait for pecan improvement, plant disease resistance genes (*R* genes) in the *C. glabra* genome were predicted using the DRAGO 2 pipeline (with default parameters) from the Plant Resistance Genes database (PRGdb 3.0) (Osuna-Cruz et al. 2018). Using the same pipeline, *R* genes were also identified in 3 other *Carya* species with assembled genomes: *C. illinoinensis*, *C. cathayensis*, and *C. sinensis*. In addition, we focused particularly on resistance to *Phylloxera*—aphid-like insects that induce gall formation in pecan. A major quantitative trait locus (QTL) associated with phylloxera resistance was identified by Lovell et al. (2021) in *C. illinoinensis*. Using the primary assembly of *C. illinoinensis* cv. ‘Lakota’ as the reference, this QTL is located on chromosome 16 (positions 1521681 to 2392040), between genes CiLak.16G012100 and CiLak.16G019000 (Lovell et al. 2021). Syntenic regions in *C. glabra* corresponding to this QTL were detected and visualized using MCScan from JCVI (v.1.2.10; Tang et al. 2024). Within these syntenic regions, putative *R* genes were identified across all 4 *C. glabra* haplotypes.

### Phylogenomic analysis of *Carya*

We used the target and probe design module of Captus (v.1.6.6; Ortiz et al. 2023) to select a subset of low-copy protein-coding genes from the coding sequence (CDS) of the fully assembled and annotated genomes (*C. cathayensis*, *C. glabra*, *C. hunanensis*, *C. illinoinensis*, *C. sinensis*, and *Juglans regia*). First, CDS were deduplicated within samples and clustered across samples using default parameters in “captusd cluster” (deduplication identity threshold of 99%, clustering identity threshold of 75%). Subsequently, “captusd select” was used to filter cluster alignments based on the following criteria: single-sequence representation across all 6 species, 35% to 65% GC content, 300 to 4,000 bp in length, a minimum of 1 parsimony-informative site up to 6% of the total length, and a maximum of 20% missing data per alignment. Representative sequences for each cluster were determined with a 91% sequence identity threshold using the “new_targets_from_alignments” script in Captus, which resulted in a target file containing 6,804 sequences that represented 6,435 loci; we named this target file Carya6435.

The assembly module of Captus (v.1.6.6; Ortiz et al. 2023) was then used to clean and assemble reads and extract and align loci data across 29 samples representing 19 species (18 *Carya* species and 1 outgroup). The read sequence data summarized in Supplementary Table 3 were downloaded from NCBI, and raw FASTQ files were cleaned using default options in “captus clean.” Cleaned reads were de novo assembled with “captus assemble” using preset “WGS” for the samples annotated as “WGS” (high-depth whole-genome sequencing) or “CAPSKIM” for the samples annotated as “CAP” (target capture) or “GSK” (genome skimming) in the phylogeny. The Carya6435 sequences were searched and extracted from the 23 samples assembled with Captus as well as from the 6 samples corresponding to fully assembled genomes with “captus extract” using a minimum sequence identity of 75% and a paralog identity tolerance of 99% to match the settings used for the creation of the targets in “captusd cluster.” The 6,435 loci were aligned across the 29 samples using default options and designating *J. regia* as the outgroup in “captus align.”

The accessory script “phylo_commands’ of Captus (v.1.6.6) was used to generate the necessary commands to estimate a phylogenetic tree for each locus with IQ-TREE (v.3.0.1; Wong et al. 2025). The resulting gene trees were used as input for coalescent species tree estimation using ASTRAL-Pro3 (v.1.23.3.6; Zhang et al. 2025).

## Results

### Statistics of sequence data

The basic statistics of the raw sequence data are summarized in Table 1. PacBio HiFi reads were generated on 2 SMRT cells, yielding a total of 79.1 gigabases (Gb) of data (44.1 Gb from 1 cell and 35.0 Gb from the other cell) (Table 1). In total, 5.3 million HiFi reads were obtained, with an average read length of 15.0 kilobases (kb). The proportions of bases with quality scores greater than 20 (Q20) and 30 (Q30) were 97.7% and 94.5%, respectively. The sequencing coverage, calculated by dividing the total number of bases by the monoploid genome size (1*x*), was 131.7× (Table 1). Given that the *C. glabra* is a tetraploid and comprises 4 haplotypes, the coverage per haplotype was therefore 32.9×.

**Table 1.** Basic statistics of the raw sequence data from *C. glabra*.

	PacBio HiFi	Omni-C	RNA-seq	
			Leaf	Axillary bud
Total bases (Gb)	79.1	39.7	24.3	22.5
Total read number (million)	5.3	264.5	160.6	148.8
Average read length (bp)	15,035.2	150.0	151.0	151.0
Coverage^a^	131.7×	66.1×	-	-

^a^Sequencing coverage was calculated by dividing the total number of bases by the assembled monoploid (1*x*) genome size (600.4 Mb for haplotype A, as described in the nuclear genome assembly section).

For Omni-C data, a total of 264.5 million reads (derived from paired-end sequencing of 132.3 million DNA fragments) were generated, and the total number of bases was 39.7 Gb (Table 1). The Q20 and Q30 quality scores were 98.7% and 96.4%, respectively. Sequencing coverage was 66.1×, corresponding to 16.5× per haplotype in the tetraploid genome.

RNAs extracted from leaf and axillary bud tissues were of high quality, with RNA integrity (RIN) scores of 7.1 and 7.2, respectively. For RNA-seq data, 161.6 million reads (from paired-end sequencing of 80.3 million fragments) were generated from the leaf tissue, and the Q20 and Q30 quality scores were 99.0% and 96.1%, respectively (Table 1). We also generated 148.8 million reads from the axillary bud tissue, and the Q20 and Q30 scores were 99.0% and 96.0%, respectively.

### Chloroplast and mitochondrial genome assembly and annotation

**Fig. 2.**
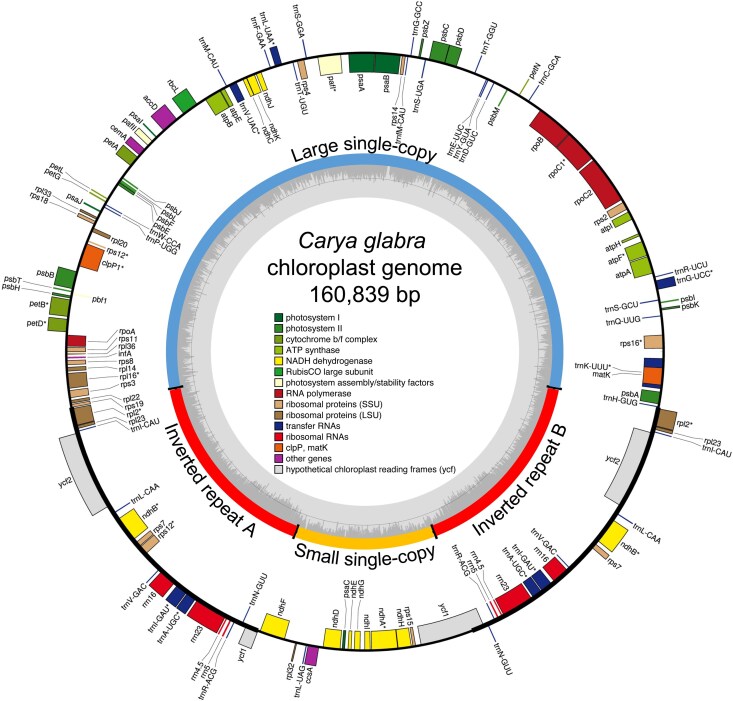
Annotated chloroplast genome of *C. glabra*. The outermost circle shows the annotated genes, color-coded according to their functional categories (legend displayed in the figure center). Genes on the inside of the circle are transcribed clockwise, whereas those on the outside are transcribed counterclockwise. Intron-containing genes are marked with an asterisk (*). The inner circle indicates the 4 structural regions of the chloroplast genome: the LSC, the SSC, and the 2 IR regions (a and b). The innermost gray graph represents the GC content, with the gray reference line marking the 50% threshold. The figure is modified from the OGDRAW output.

The chloroplast genome of *C. glabra* is 160,839 bp in length and has the typical quadripartite structure (Fig. 2). The genome is composed of a pair of inverted repeat (IR) regions (i.e. IRA and IRB; 26,006 bp in length for each region), a large single-copy (LSC) region (90,041 bp), and a small single-copy (SSC) region (18,786 bp) (Fig. 2). A total of 113 unique genes, including 79 protein-coding genes, 30 tRNA genes, and 4 rRNA genes, were annotated (Fig. 2). A detailed list of these genes, along with their functional categories and genomic locations, is provided in Supplementary Table 4. The GC contents of LSC, SSC, and IR regions were 33.7%, 29.9%, and 42.6%, respectively.

**Fig. 3.**
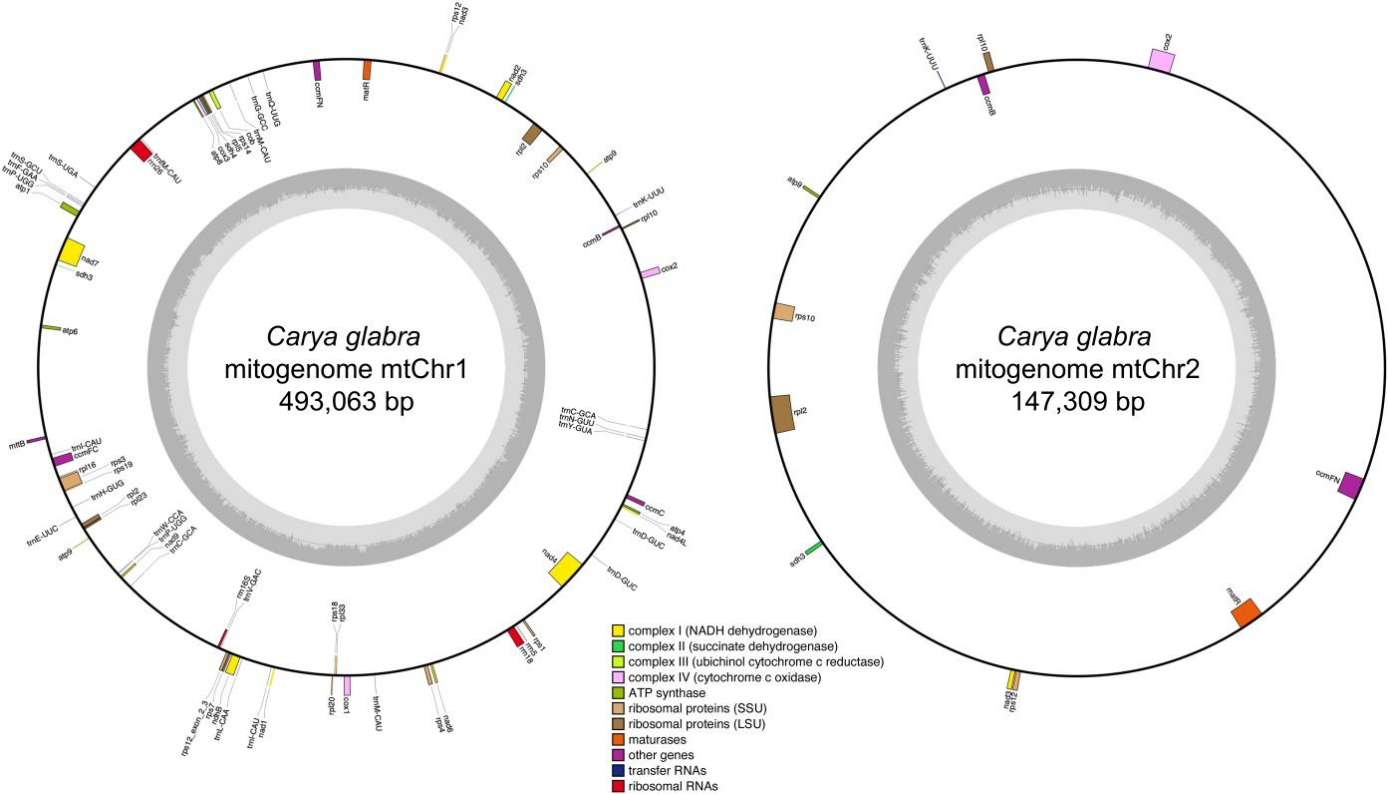
Annotated mitochondrial genome of *C. glabra* shown as 2 conformations labeled mtChr1 and mtChr2. The outermost circle shows the annotated genes, color-coded according to their functional categories (legend displayed at bottom center). Genes on the inside of the circle are transcribed clockwise, whereas those on the outside are transcribed counterclockwise. The innermost gray graph represents the GC content, with the gray reference line marking the 50% threshold. Chromosomes are not drawn to scale. The figure is modified from the OGDRAW output.

The 2 mitochondrial chromosomes are 493,063 and 147,309 bp in length (Fig. 3). The larger chromosome (mtChr1) also presents a quadripartite structure where 2 inverted repeats (mtIR) of 2,760 bp intercalate a small single-copy (mtSSC) region (135,915 bp) and a large single-copy (mtLSC) region (351,628 bp). The smaller chromosome (mtChr2) is mostly redundant with mtChr1, consisting of one of the mtIRs, the entire mtSSC, 1,795 bp of the mtLSC, and a unique segment of 6,839 bp. A total of 42 protein-coding genes, 23 tRNA genes, and 3 rRNA genes were annotated in the mitochondrial genome (Supplementary Table 5). From these, 15 were annotated as functional plastome-derived genes (5 protein-coding genes and 10 tRNA genes) (Supplementary Table 5). We additionally identified 15 nonfunctional plastome genes: 6 were complete but contained premature stop codons, and 9 were only fragmentary. All plastome-derived genes were located inside several sequence segments with varying lengths and degrees of conservation, as measured by their sequence identity to the chloroplast assembly (Supplementary Table 6). Most notably, 2 large segments contained multiple functional plastome genes: The first segment (15,031 bp, 99.2% identity) contained *trnA-UGC*, *trnI-CAU*, *trnL-CAA*, and *trnV-GAC* genes; and the second segment (2,137 bp, 84.3% identity) contained *psaJ*, *rpl20*, and *rpl33* genes (Supplementary Table 6).

### Nuclear genome profiling

**Fig. 4.**
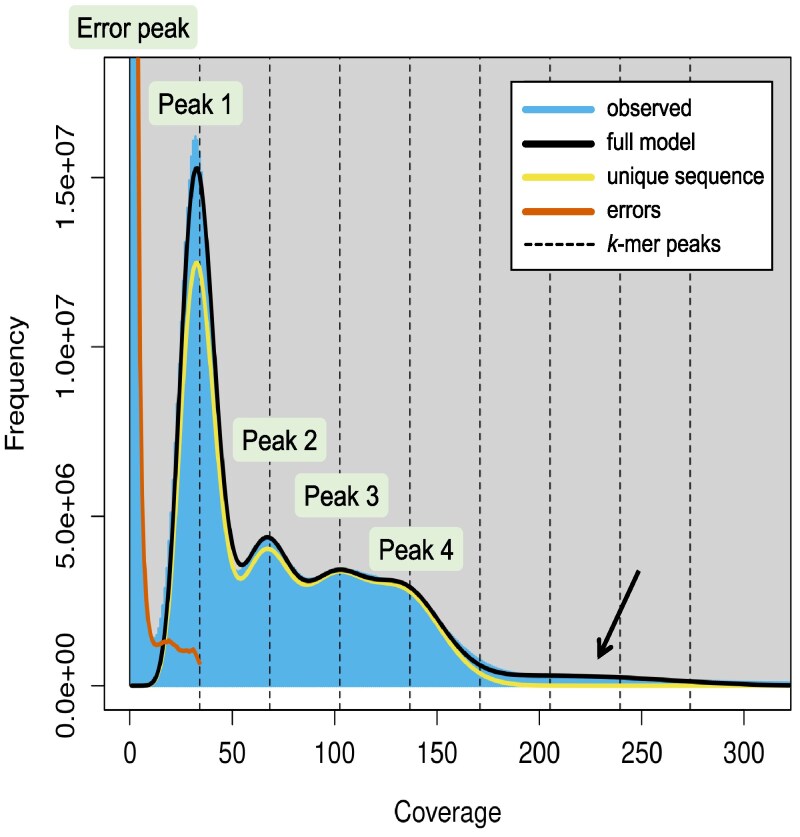
*k*-mer spectrum of *C. glabra*. The plot illustrates the distribution of *k*-mer frequencies (i.e. counts of unique *k*-mers; *y* axis) across different coverage depths (*x* axis) in the entire HiFi dataset. The leftmost error peak, representing the large number of low-coverage unique *k*-mers, results from sequencing errors. Peaks 1, 2, 3, and 4 correspond to *k*-mers present in 1, 2, 3, and 4 copies, respectively, within the tetraploid genome. The coverages for peaks 1, 2, 3, and 4 are 34.2×, 68.4×, 102.6×, and 136.8×, respectively. The high-coverage “hump,” indicated by the arrow, represents *k*-mers derived from repetitive regions. *k*-mer size: 21. The figure is modified from the GenomeScope 2.0 output.

Based on *k*-mer frequency analysis of the unassembled HiFi reads, GenomeScope 2.0 estimated the monoploid genome size as 515.4 Mb, with a heterozygosity value of 4.9% and repetitive sequences accounting for 38.5% of the genome. The frequencies of the heterozygous forms *aaab* and *aabb* were 3.2% and 1.4%, respectively. The resulting *k*-mer spectrum is shown in Fig. 4. The 4 major peaks, corresponding to *k*-mers present in 1 to 4 copies, are characteristic of an autotetraploid genome (Ranallo-Benavidez et al. 2020).

### Nuclear genome assembly and annotation

The initial unitig assembly generated by hifiasm comprised 2,856 unitigs with an N50 of 7.5 Mb. A dot plot comparing this unitig assembly with 1 set of chromosomes from the *C. illinoinensis* genome revealed that each *C. illinoinensis* region corresponded to 4 unitigs, confirming the tetraploid nature of the *C. glabra* genome and indicating that the unitig assembly incorporated genomic sequences from all 4 haplotypes (Supplementary Fig. 1). The complete BUSCO score for the unitig assembly was 98.9%, consisting of 1.0% single-copy and 97.9% duplicated BUSCOs; the high proportion of complete and duplicated BUSCOs reflects that sequences from all haplotypes were represented in the assembly.

Next, the unitigs were scaffolded by YaHS using the Omni-C data. Based on the hic_qc analysis, the Omni-C library was considered “sufficient,” showing high proportions of long-distance and interunitig contacts (Supplementary Table 7). The initial YaHS scaffolding resulted in 2,584 scaffolds with an N50 of 36.9 Mb, including 62 scaffolds with a length longer than 10 Mb. Examination of the Hi-C contact map, along with the dot plot comparing the *C. illinoinensis* genome with the initial YaHS scaffolds, revealed several scaffolding errors, including 2 misjoins and an inversion error, which were corrected manually using Juicebox (Supplementary Fig. 2). In addition, Juicebox was used to reorient several scaffolds to match the chromosome orientations of *C. illinoinensis*.

**Fig. 5.**
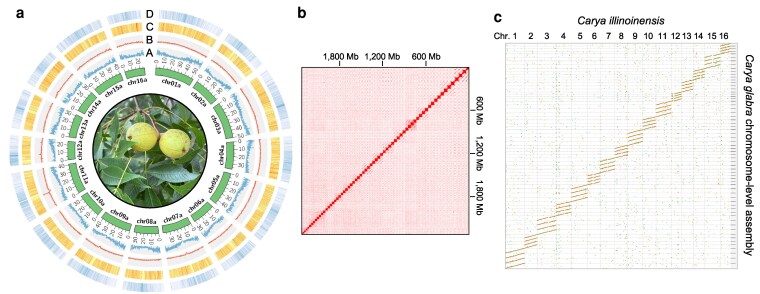
The chromosome-level assembly of the *C. glabra* (4*x*) nuclear genome. a) Circos plot of the 16 chromosomes from haplotype A of the *C. glabra* genome. The unit of the chromosome length is Mb. The densities of various genomic features in 100-kb sliding windows across the chromosomes are shown on 4 tracks (A, genes; B, transposons; C, *copia*; D, *gypsy*). b) The Hi-C contact map of the nuclear genome assembly. c) The dot plot comparing 1 set of chromosomes from *C. illinoinensis* (2*x*) and the 4 sets of chromosomes from *C. glabra*.

After manual curation, the 64 longest scaffolds in the final assembly (each >10 Mb) accounted for 94.8% of the total assembled sequences (2,319.4 Mb out of 2,445.8 Mb) and corresponded to the expected chromosome number of the *C. glabra* genome (Fig. 5). Hereafter, we refer to these 64 scaffolds as pseudochromosomes (or simply chromosomes for brevity). Each pseudochromosome was named according to its syntenic similarity with the *C. illinoinensis* genome based on the dot plot (Fig. 5c) and was assigned to haplotypes (A through D) based on descending length. It is important to note that this haplotype assignment is artificial and does not necessarily reflect true biological haplotypes (see Materials and methods). The monoploid genome (1*x*) sizes for haplotypes A, B, C, and D were 600.4, 585.2, 574.3, and 559.4 Mb, respectively (Table 2). In addition, the complete BUSCO scores for the assembled genomes were 97.8%, 97.6%, 96.8%, and 95.4% for haplotypes A, B, C, and D, respectively (Table 2). Detailed statistics for each chromosome are provided in Supplementary Table 8.

**Table 2.** Assembly statistics and genomic features of the *C. glabra* genome and other published genomes of *Carya* species.

Genome statistics	*C. glabra* (4*x*)				*C. illinoinensis* (2*x*)^a^	*C. sinensis* (2*x*)	*C. cathayensis* (2*x*)
	Hap. A	Hap. B	Hap. C	Hap. D			
Monoploid (1*x*) genome size (Mb)	600.4	585.2	574.3	559.4	674.3	623.2	698.1
N50 (Mb)	39.6	37.7	36.8	36.2	44.7	38.9	43.5
Repeat sequences (%)	55.0	54.4	54.0	53.8	49.7	43.5	50.1
Predicted protein-coding genes	30,947	31,087	30,369	30,110	32,267	35,370	36,722
Complete BUSCO (%) assembly	97.8	97.6	96.8	95.4	98.1	96.9	97.0
Complete BUSCO (%) annotation	97.7	97.1	96.5	94.9	96.3	94.8	95.8
Genes shared with *C. glabra* (%)^b^	…				91.1	91.0	90.9
Reference	Current work				Lovell et al. (2021)	Zhang et al. (2024b)	

Hap., haplotype.

^a^The statistics are from *C. illinoinensis* cv. ‘Pawnee’.

^b^A gene from the focal species is considered shared with *C. glabra* if it belongs to an orthogroup that includes 1 or more *C. glabra* genes; see Materials and methods for details.

Repetitive sequences accounted for the majority of the *C. glabra* genome (Table 2; Supplementary Table 9). In haplotypes A, B, C, and D, 55.0%, 54.4%, 54.0%, and 53.8% of the genomic sequences were classified as repetitive regions, respectively (Table 2). Specifically, retrotransposons comprised 24.7% to 27.2% of the genome across the 4 haplotypes, and DNA transposons represented 19.4% to 21.5% of the genome (Supplementary Table 9). In addition, simple repeats (duplications of short DNA motifs; microsatellites) accounted for 1.2% to 1.3% of the genome.

For protein-coding gene prediction, several BRAKER3 settings were tested using the haplotype A genome as the reference (Supplementary Table 2). The ratio of monoexonic to multiexonic genes ranged from 0.24 to 0.28 across all settings (Supplementary Table 2), which falls within the expected range reported for other plant genomes (Vuruputoor et al. 2023). The combination that used RNA-seq data from *C. glabra* and protein evidence from 14 model species—followed by filtering out gene models ≤50 amino acids—produced the highest BUSCO score (97.7%) (Supplementary Table 2). Therefore, the same setting was used to annotate the genes from haplotypes B, C, and D.

A total of 30,947 genes were predicted for haplotype A, with an average CDS length of 1,241 bp (Table 2; Supplementary Table 10). For haplotypes B, C, and D, the number of predicted protein-coding genes ranged from 30,110 to 31,087 (Table 2). The average CDS length ranged from 1,239 to 1,254 bp (Supplementary Table 10). All haplotypes had an average of 5.0 exons per gene, and the average gene length varied between 4,364 and 4,460 bp (Supplementary Table 10).

TRAPID annotation assigned gene family information to 94.3% of the predicted genes in haplotype A, with 79.7% and 86.3% of genes annotated with Gene Ontology (GO) terms and protein domains, respectively (Supplementary Table 10). The core gene family completeness score in TRAPID was 0.982, exceeding the conservation threshold of 0.9, further supporting the high completeness of the predicted gene models. Similarly, haplotypes B, C, and D showed high annotation rates: 93.9% to 94.7% of genes were assigned to gene families, and 85.9% to 86.5% were annotated with protein domains (Supplementary Table 10). All haplotypes also exhibited high BUSCO completeness scores based on the annotated genes, ranging from 94.9% to 97.7% (Table 2).

### Comparative genomic analysis

**Fig. 6.**
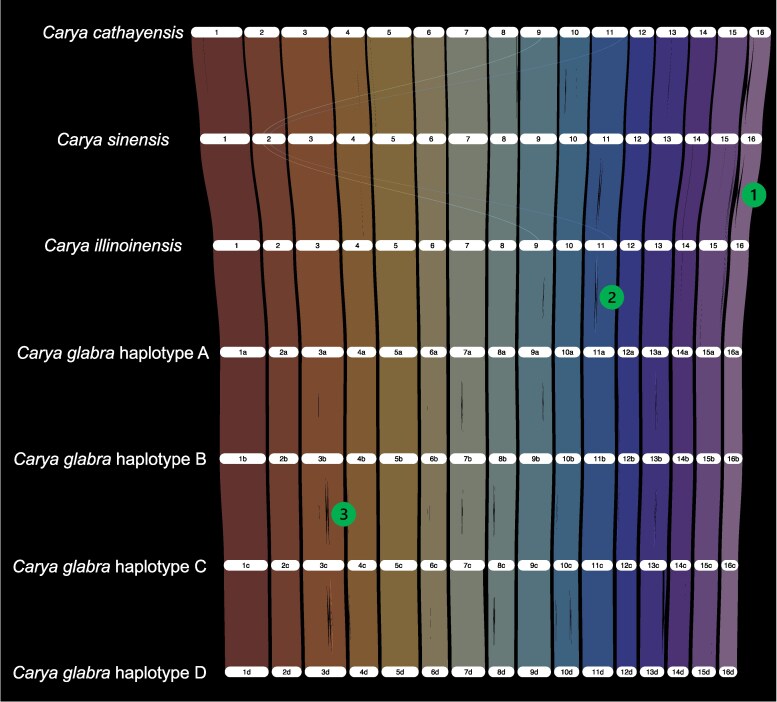
Syntenic map (riparian plot) of homologous regions among the 4 haplotypes of *C. glabra* and the haploid genomes of *C. cathayensis*, *C. sinensis*, and *C. illinoinensis*. The chromosomes are scaled by gene rank order. Among the structural variants identified, 3 are highlighted: green circle 1 marks an inversion on chromosome 16 between *C. sinensis* and *C. illinoinensis*; green circle 2 indicates an inversion between *C. illinoinensis* and haplotype A of *C. glabra* on chromosome 11; and an inversion between *C. glabra* haplotypes B and C on chromosome 3 is indicated by green circle 3.

Synteny analysis was performed among the 4 haplotypes of *C. glabra* and the haploid genomes of *C. cathayensis*, *C. illinoinensis*, and *C. sinensis*, revealing high overall collinearity among the genomes (Fig. 6). However, several structural variants were also identified. For example, an inversion on chromosome 16 was detected between the *C. sinensis* and *C. illinoinensis* genomes (indicated by green circle 1 in Fig. 6). Another inversion on chromosome 11 was observed between *C. illinoinensis* and all 4 haplotypes of *C. glabra* (green circle 2); this inversion was also evident in the corresponding dot plot (Fig. 5c). Furthermore, structural variation was found among the 4 *C. glabra* haplotypes. For instance, the synteny analysis showed an inversion on chromosome 3 unique to haplotype C, which was also detected in the dot plot (Fig. 5c; green circle 3 in Fig. 6).

OrthoFinder analysis showed that, of the 32,267 annotated *C. illinoinensis* genes, 91.1% were shared with *C. glabra*; that is, 91.1% occurred in an orthogroup containing 1 or more *C. glabra* genes (Table 2). Similarly, 91.0% and 90.9% of genes in *C. sinensis* and *C. cathayensis*, respectively, were shared with *C. glabra*.

### Disease resistance genes in *C. glabra*

Plant disease resistance genes, i.e. *R* genes, across the 4 haplotypes were predicted. Specifically, we focused on 4 major classes of *R* genes: CNL [containing the coiled-coil domain, the nucleotide-binding site {NBS} domain, and the leucine-rich repeat {LRR} domain], TNL (containing the Toll-interleukin receptor-like domain, the NBS domain, and the LRR domain), RLP [receptor-like protein, containing the transmembrane {TM} domain and the LRR domain], and RLK (receptor-like kinase, containing the TM domain, the LRR domain, and the kinase domain). In haplotype A, we identified 625 putative *R* genes from these 4 classes, including 56 CNL, 39 TNL, 214 RLP, and 316 RLK class genes (Supplementary Table 11). For haplotypes B, C, and D, 638, 655, and 608 putative *R* genes were annotated, respectively (Supplementary Table 11). In addition, we identified 724, 685, and 800 putative *R* genes in the primary assemblies of *C. illinoinensis*, *C. sinensis*, and *C. cathayensis*, respectively (Supplementary Table 11).

**Fig. 7.**
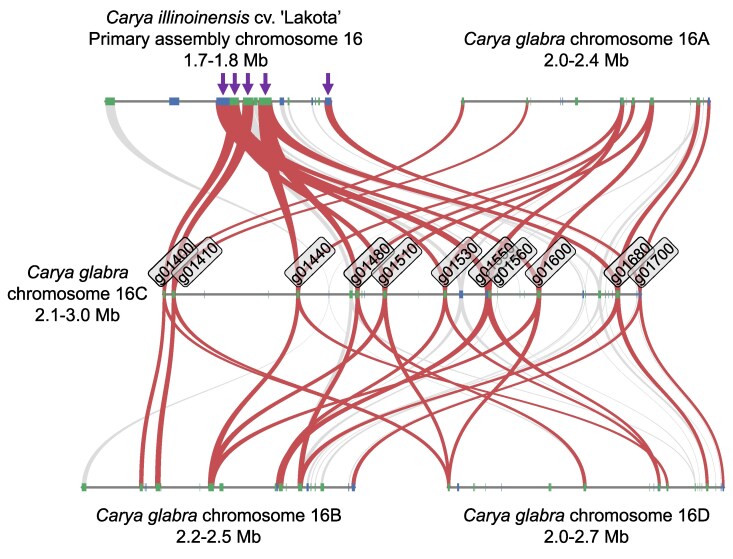
Synteny between *C. illinoinensis* cv. “Lakota” and the 4 *C. glabra* haplotypes at the major QTL associated with phylloxera resistance. QTL mapping in *C. illinoinensis* by Lovell et al. (2021) identified a single large QTL peak on chromosome 16. Within this QTL on the primary assembly of *C. illinoinensis* cv. ‘Lakota’, 5 putative plant disease resistance genes (*R* genes) containing the LRR domain were annotated (indicated by arrowheads). In the corresponding syntenic region of *C. glabra*, chromosome 16C contains 11 putative *R* genes—the highest count among the 4 haplotypes—with each gene labeled by name. The syntenic regions on chromosomes 16A, 16B, and 16D contain 8, 10, and 8 *R* genes, respectively. Syntenic gene pairs are connected by the ribbons, with those linking to the 11 *R* genes on *C. glabra* chromosome 16C highlighted in red. Note that not all *R* genes on chromosomes 16A, 16B, and 16D are reciprocal best hits with *R* genes on chromosome 16C; therefore, these are not connected with red ribbons in the plot. Genes are depicted as boxes, with blue representing genes on the positive strand and green representing genes on the negative strand. Chromosome segments are not drawn to scale.

The syntenic regions in *C. glabra* corresponding to the major QTL for phylloxera resistance in *C. illinoinensis* were identified on chromosome 16 (Fig. 7). Within these syntenic regions, 8, 10, 11, and 8 *R* genes were detected in haplotypes A, B, C, and D, respectively (Fig. 7; Supplementary Table 12). Syntenic gene pairs between the 5 *R* genes annotated in the primary assembly of *C. illinoinensis* cv. ‘Lakota’ and their counterparts in *C. glabra* were highlighted in the synteny plot (Fig. 7). Among the 37 *C. glabra R* genes located in these syntenic regions, 30 belong to the TNL class, while 3 and 4 belong to the RLP and RLK classes, respectively (Supplementary Table 12).

### *Carya* phylogenomics

**Fig. 8.**
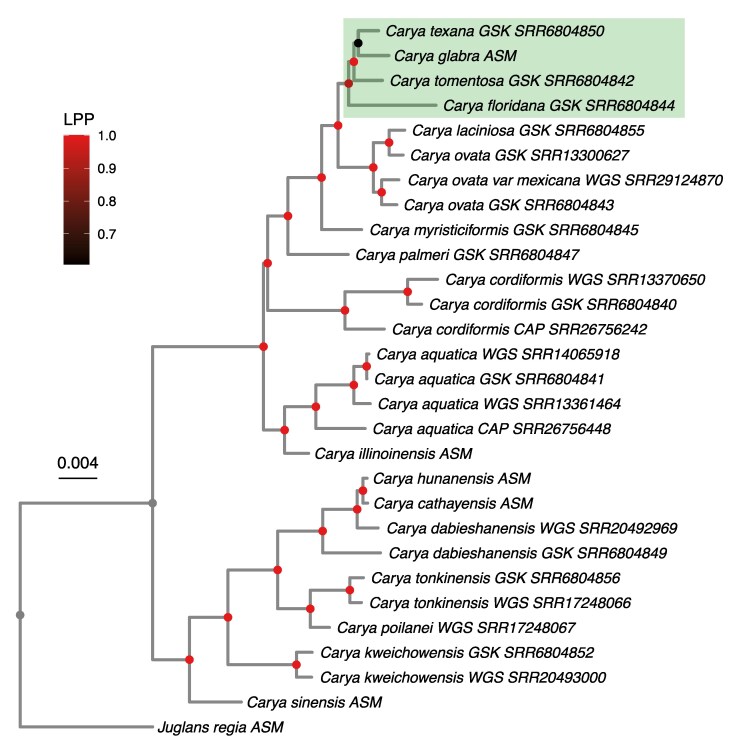
Coalescent species tree for *Carya* estimated with ASTRAL-Pro3 based on 6,435 nuclear gene trees estimated with IQ-TREE3. The green box highlights the tetraploid clade. LPP, local posterior probability; GSK, genome skimming; ASM, fully assembled genome; CAP, target capture; WGS, high-depth whole-genome sequencing. Branch lengths in substitution-per-site units, calculated by CASTLES-Pro in ASTRAL-Pro3.

Our phylogenomic analyses based on 6,435 low-copy nuclear DNA regions provided a well-resolved phylogeny that included all but 2 species of the genus *Carya* (i.e. *Carya luana* and *Carya pallida*) (Fig. 8). The North American species of *Carya* form a well-supported clade sister to the clade of Asian species. Significantly, the 4 species from North America known to be tetraploid (*Carya floridana*, *C. tomentosa*, *C. texana*, and *C. glabra*) form a distinct clade. The diploids *Carya laciniosa* and *Carya ovata* are the immediate sister group to this tetraploid clade. Branch lengths within the tetraploid clade are short and support values low; *C. floridana* followed by *C. tomentosa* are recovered as subsequent sisters to a clade of *C. texana* and *C. glabra.*

## Discussion

### *C. glabra* organellar genomes

The chloroplast genome size in Juglandaceae ranges from 158,223 to 161,713 bp (Liu et al. 2025). Three *C. glabra* chloroplast genomes have been published to date (Luo et al. 2021; Xi et al. 2022; Liu et al. 2025), with sizes ranging from 160,645 to 160,652 bp. In the present study, the assembled chloroplast genome of *C. glabra* is 160,839 bp in length (Fig. 2), very similar to the published *C. glabra* chloroplast genomes and within the size range observed across species from other Juglandaceae.

A total of 109, 113, and 114 unique genes were annotated in previously published *C. glabra* chloroplast genomes with NCBI accession numbers OR099205, NC_067504, and BK061156, respectively. In our study, 113 unique genes were identified, including 79 protein-coding genes, 30 tRNA genes, and 4 rRNA genes (Fig. 2; Supplementary Table 4). The additional gene reported in accession BK061156 is *ycf15*, a functionally uncharacterized gene that is also absent from the well-annotated *Nicotiana tabacum* chloroplast genome (NC_001879). Through manual curation, we identified several misannotated and missing genes in previously reported *C. glabra* chloroplast genomes (summarized in Supplementary Table 13). For example, additional copies of tRNA genes *trnA-UGC* and *tnrM-CAU* were misannotated in BK061156; 2 protein-coding genes, *atpB* and *rpoB*, were missing from OR099205; and the first exons of *petB*, *petD*, and *rpl16* were absent from NC_067504. All such potential annotation errors were manually corrected in the present study. Together, these results indicate that although several *C. glabra* chloroplast genomes have been published, our assembly and annotation represent the most complete and accurate version to date.

Compared to chloroplast genomes, the reports of the assembly of plant mitochondrial genomes are few, primarily due to the high structural complexity of the mitogenome in plants (Palmer and Herbon 1988; Møller et al. 2021; Wu et al. 2022; Wang et al. 2024). Wang et al. (2024) reported that approximately 13,000 complete chloroplast genomes have been assembled, compared with only 673 complete mitochondrial genomes. We have found published mitochondrial genomes from 3 species in Juglandaceae, which show substantial variation in structure and gene content. The *J. regia* (Juglandaceae) mitogenome consists of 3 circular chromosomes and includes 39 protein-coding genes, 47 tRNA genes, and 5 rRNA genes (Ye et al. 2024). The *Juglans mandshurica* mitochondrial genome includes 2 chromosomes and has 38 protein-coding genes, 20 tRNA genes, and 3 rRNA genes (Su et al. 2023). Comparative genomic analysis between *J. regia* and *J. mandshurica* identified 66 homologous fragments, including 28 collinear blocks and 38 inversions, reflecting extensive structural rearrangements (Ye et al. 2024). Chen et al. (2024) assembled the first mitochondrial genome of *C. illinoinensis*: The single circular genome is 495.2 kb in length and contains 37 protein-coding genes, 24 tRNA genes, and 3 rRNA genes. In *C. glabra*, the mitogenome includes 2 chromosomes (493.1 and 147.3 kb in length), and we identified 42 protein-coding genes, 23 tRNA genes, and 3 rRNA genes (Fig. 3; Supplementary Table 5). Although mitogenomes are generally highly variable, the *C. glabra* mitochondrial genome is broadly comparable with other published Juglandaceae mitogenomes. For example, of the 69 mitochondrial genes identified in *C. glabra*, *C. illinoinensis*, and *J. mandshurica*, 54 genes (78.3%) are shared among all 3 species (Supplementary Fig. 3; Supplementary Table 14).

The varying sizes and identities of the plastome segments detected in the *C. glabra* mitochondrial genome suggest multiple transfer events occurring at different times (Supplementary Table 6). In addition, 6 of the 7 mitochondrial genes unique to *C. glabra*, and absent from *C. illinoinensis* and *J. mandshurica*, are derived from these plastome segments (Supplementary Table 14). In future studies, it would be interesting to compare these transferred segments with other congeneric chloroplast and mitochondrial genomes.

### Nuclear genomes in *Carya*

We assembled and annotated the first nuclear genome of *C. glabra* (Fig. 5). The assembly is chromosome level and haplotype resolved, representing the first assembled polyploid genome in the genus (Fig. 5). Furthermore, GenomeScope 2.0 predicted that *C. glabra* is an autotetraploid based on the pattern of nucleotide heterozygosity levels: The frequency of the heterozygous *aaab* genotype was higher than that of the *aabb* genotype (3.2% vs 1.4%), a pattern reported to be characteristic of autopolyploids (Ranallo-Benavidez et al. 2020). Additionally, the *k*-mer spectrum showing 4 major peaks (Fig. 4), along with the high similarity among the 4 copies of each chromosome compared to the *C. illinoinensis* (2*x*) genome based on the dot plot (Fig. 5c), further support that *C. glabra* is an autotetraploid.

In terms of genomic composition, 53.8% to 55.0% of the *C. glabra* genome consists of repetitive sequences, with slight variation among haplotypes (Table 2). Similar, but lower, proportions of repetitive content have been reported in other *Carya* species. Lovell et al. (2021) found that 49.7% of the *C. illinoinensis* genome is repetitive sequences, and Zhang et al. (2024b) reported repeat fractions in the genomes of *C. sinensis* (43.5%) and *C. cathayensis* (50.1%) (Table 2).

We predicted more than 30,000 protein-coding genes for each *C. glabra* haplotype (Table 2). BUSCO completeness scores were high across all haplotypes, with haplotype A having a BUSCO score of 97.7%. The number of genes predicted in *C. glabra* is broadly comparable to those reported for other *Carya* species (Table 2). Lovell et al. (2021) annotated 32,267 genes in *C. illinoinensis*, and Zhang et al. (2024b) identified 35,370 and 36,722 genes in *C. sinensis* and *C. cathayensis*, respectively (Zhang et al. 2024b). OrthoFinder analyses showed that 91.1%, 91.0%, and 90.9% of genes in *C. illinoinensis*, *C. sinensis*, and *C. cathayensis* were shared with *C. glabra*, respectively (Table 2).

Several nonmutually exclusive factors may explain the differences in gene count among *Carya* genomes. First, the annotation pipeline can affect the number of predicted genes. Weisman et al. (2022) found that applying different annotation methods to the same genome can lead to the identification of genes unique to each method. In this study, we used BRAKER3 for gene annotation, whereas PASA (Haas et al. 2003) and FGENESH (Salamov and Solovyev 2000) were used to annotate the *C. illinoinensis* genome (Lovell et al. 2021). Zhang et al. (2024b) used PASA, AUGUSTUS (Stanke et al. 2006), and GeneWise (Birney et al. 2004) to annotate the *C. sinensis* and *C. cathayensis* genomes. Second, the diversity and number of tissues represented in the RNA-seq data can affect annotation completeness, and sampling from multiple tissues is recommended (Salzberg 2019; Kress et al. 2022; Vuruputoor et al. 2023). Our annotations were supported by RNA-seq data from 2 tissues (leaf and axillary bud), whereas Lovell et al. (2021) used RNA-seq data from a larger number of tissues, including leaf, catkin, and dormant and swelling buds. Lastly, the lower gene count in *C. glabra* may reflect its polyploid nature. Genome fractionation and gene loss are common following polyploid formation (Langham et al. 2004; Leitch and Bennett 2004; Freeling 2009; Soltis et al. 2015; Van de Peer et al. 2017; Wendel et al. 2018), although fractionation as originally defined (Freeling 2009) cannot occur in an autopolyploid that lacks parental subgenomes. Indeed, the relatively smaller monoploid (1*x*) genome size of *C. glabra* (e.g. 600.4 Mb for haplotype A and smaller for the other haplotypes), compared with that of diploid *Carya* species (e.g. 674.3 Mb for *C. illinoinensis*), may result from gene loss following polyploidy in *C. glabra*.

In summary, the *C. glabra* genome assembly and annotation presented in this study are of high quality, with metrics comparable to, or surpassing (based on the BUSCO completeness score; Table 2), published genomes from other *Carya* species.

### Potential practical applications of the *C. glabra* genome assembly

The *C. glabra* genome assembly provides a valuable resource for identifying candidate genes that may facilitate breeding programs in pecan (*C. illinoinensis*) and Chinese hickory (*C. cathayensis*). Disease resistance is a key target in pecan breeding (Bock et al. 2016; Bhattarai et al. 2025), and crop wild relatives provide valuable genetic resources for crop improvement (Bohra et al. 2022). Notably, we identified over 600 disease resistance genes (*R* genes) in each haplotype of *C. glabra* (Supplementary Table 11). A slightly higher number of *R* genes has been identified in other *Carya* species: *C. illinoinensis*, *C. sinensis*, and *C. cathayensis* have 724, 685, and 800 *R* genes, respectively (Supplementary Table 11). We focused particularly on a genomic region syntenic to a major QTL associated with phylloxera resistance in *C. illinoinensis*. Several aphid-like insects from the genus *Phylloxera* infect pecan and induce gall formation, which can cause defoliation and significantly reduce yield (Hedin et al. 1985; Andersen and Mizell 1987). Lovell et al. (2021) identified a single major QTL underlying this trait, and several candidate *R* genes containing LRR domains were annotated within this QTL. In the syntenic region in *C. glabra*, we identified 8, 10, 11, and 8 *R* genes in haplotypes A, B, C, and D, respectively (Fig. 7; Supplementary Table 12). These candidate genes provide an additional genetic resource that could facilitate engineering efforts to improve phylloxera resistance in pecan.

Polyploidy plays an important role in plant breeding, by providing genome buffering, increased allelic diversity and heterozygosity, and novel phenotypic variation (Udall and Wendel 2006; Sattler et al. 2016), and polyploids often exhibit an advantageous stress response relative to diploids (Bomblies 2020; Fox et al. 2020; Van de Peer et al. 2021; Tossi et al. 2022). Future studies examining stress response in *C. glabra* and its closely related diploid species (e.g. *C. palmeri* and *C. illinoinensis*) could provide valuable insights into the effect of polyploidy on stress tolerance in *Carya*—information that may inform future strategies for improving pecan and Chinese hickory.

### Phylogenomics of *Carya*

We provide a well-resolved and generally well-supported phylogenetic tree for *Carya* based on near-complete taxon sampling and numerous nuclear DNA regions (Fig. 8). This is the most comprehensive nuclear-based tree produced to date for the genus *Carya*. North American and Asian species each form well-supported sister clades. Within the North American clade, the 4 tetraploid species (*C. floridana*, *C. tomentosa*, *C. texana*, and *C. glabra*) form a distinct clade, a pattern also recovered by Huang et al. (2019).

Within the tetraploid North American clade, branch lengths are short, suggesting a recent, rapid divergence (Fig. 8). Importantly, among the North American tetraploids, *C. floridana* followed by *C. tomentosa* are recovered as subsequent sisters to a clade of *C. texana* and *C. glabra*. Although published plastome sequence data indicate that other close relatives of *C. glabra* include diploid *Carya palmeri* and some but not all populations of pecan (also diploid), *C. illinoinensis* (Xi et al. 2022), our nuclear phylogenomic data do not recover such relationships. Instead, *C. illinoinensis* is well separated from the North American tetraploids, whereas *C. palmeri* is several branches removed from the tetraploid clade.

Our data suggest a single origin of polyploidy in the North American clade—an event that preceded the origin of *C. floridana*, *C. tomentosa*, *C. texana*, and *C. glabra*. We recovered the diploids *C. palmeri* followed by *Carya myristiciformis*, and then a clade of *C. laciniosa* plus *C. ovata* as subsequent sisters to the North American tetraploids. As immediate sisters to the North American tetraploids, *C. laciniosa* plus *C. ovata* (or their ancestors) may have played a role in the origin of the tetraploid clade. The close plastid relationship of *C. illinoinensis* and *C. palmeri* to some of the tetraploids may reflect more recent hybridization of diploids to members of the polyploid clade. Our nuclear phylogenomic framework allows for the testing of these and other evolutionary hypotheses.

### Genome assembly and annotation as tools for conservation and teaching genomics

McCarty Woods is a 2.9-acre (11,735.9 m^2^) designated Conservation Area located at the heart of the UF campus (Fig. 1b). Representing part of the southernmost extent of deciduous forest in eastern North America, McCarty Woods contains more than 100 native plant species, including *C. glabra* (Sharman 2024). Although designated as a Conservation Area, McCarty Woods' central location on the UF campus has made it a recurring target for development. In 2021, a campaign led by botanists at the Florida Museum of Natural History as well as students and community members successfully halted proposed development plans, and efforts to advocate for long-term protection and restoration of the Woods are ongoing.

In collaboration with the ACTG project, the McCarty Woods Genome Project was launched in 2024 (Sharman 2024). By sequencing the first genomes of iconic trees growing in the Woods, the project aims to “immortalize” these individuals and provide reference genomes that will guide future research and applications involving these species. These genomic resources strengthen the case for preserving the Conservation Area status for McCarty Woods and underscore its significant value for research and education. The reference genome of *C. glabra* presented in this study represents the first genome produced by the McCarty Woods Genome Project, with others in progress (e.g. *Quercus michauxii*).

A CURE class was offered at UF in Spring 2025 as part of the McCarty Woods Genome Project (Fig. 1c). Teaching materials and data analysis pipelines from the ACTG project (Harkess 2022; Yocca et al. 2024; Zhang et al. 2024a) were incorporated into the course, providing undergraduate students with hands-on experience in genome assembly and annotation of *C. glabra*. By combining real-world data with active learning, the course engaged students from 8 departments—Biology, Biomedical Engineering, Chemistry, Computer and Information Science and Engineering, English, Entomology and Nematology, Mechanical and Aerospace Engineering, and Statistics—and emphasized programming, collaboration, critical thinking, and scientific writing. Bioinformatic code generated through the course is publicly available on GitLab (https://gitlab.com/shengchenshan/bot4935-plant-genome-assembly-and-annotation), and lecture slides are available on Zenodo (https://doi.org/10.5281/zenodo.17969442). In summary, the course provided students insight into the process of scientific research and the role of genomics in biological sciences, highlighting the value of genome assembly and annotation in training the next generation of biological scientists and bioinformaticians.

### Future directions

The *C. glabra* nuclear genome assembly provides an important tool for investigating the roles of polyploidy and hybridization in genome evolution in *Carya*. Several intriguing evolutionary questions remain. When did *C. glabra* undergo the most recent whole-genome duplication? Phylogenetic studies suggest that its closest relative is *C. texana*, which is also a tetraploid (Huang et al. 2019; Xi et al. 2022). Did these 2 species share an ancestral polyploidization event prior to divergence, or did they experience independent whole-genome duplication events? If the latter is the case, what is the diploid ancestor of *C. glabra*? Are there undetected diploid populations of *C. glabra*? What environmental factors may have contributed to the success of genome doubling in these lineages?

The possibility of gene flow between *C. glabra* and pecan (*C. illinoinensis*), which is a diploid, also merits investigation. Plastome-based phylogenetic analyses have shown that *C. glabra* is closely related to a specific *C. illinoinensis* cultivar, ‘87MX3-2.11’ (Xi et al. 2022). If introgression involving *C. glabra* and pecan occurred, it may provide novel opportunities for pecan breeding and the potential transfer of beneficial traits from *C. glabra* into this economically important crop.

## Supplementary Material

jkag135_Supplementary_Data

## Data Availability

Raw data generated in this project, including PacBio HiFi, Omni-C, and RNA-seq, are deposited in NCBI under BioProject PRJNA1373287. The 4 haplotypes of the nuclear genome assembly are available under BioProject PRJNA1376128–PRJNA1376131. The nuclear genome annotation and organellar genomes are available at Zenodo (https://doi.org/10.5281/zenodo.17969322). The chloroplast genome and mitochondrial genome chromosomes 1 and 2 have been submitted to GenBank (accession numbers PZ454095, PZ454249, and PZ454250, respectively). All codes and scripts are available at https://gitlab.com/shengchenshan/bot4935-plant-genome-assembly-and-annotation. The file listing the plant disease resistance genes (*R* genes) annotated from the haplotype A of the *C. glabra* genome is available on GSA FigShare (https://doi.org/10.25387/g3.30698447). Supplemental material available at *G3* online.
