# Supplementary material for: A haplotype-resolved, chromosome-scale genome assembly and annotation for Carya glabra (pignut hickory; Juglandaceae)
Source: G3 (Bethesda). 2026 May 20;16(8):jkag135. doi: 10.1093/g3journal/jkag135 (PMC13439944; doi:10.1093/g3journal/jkag135)
Supplement: Supplementary file 1 [file jkag135_supplementary_data.docx]

Supplementary materials

for

A haplotype-resolved, chromosome-scale genome assembly and annotation for *Carya glabra* (pignut hickory; Juglandaceae)

Fig. S1. Dot plot comparing one set of chromosomes from *Carya illinoinensis* (2*x*) with the unitig assembly of *Carya glabra* (4*x*).

Fig. S2. Manual curation of the YaHS scaffolding output using Juicebox.

Fig. S3. Venn diagram showing shared and unique mitochondrial genes among *Carya glabra*, *Carya illinoinensis*, and *Juglans mandshurica*.

Table S1. Protein evidence used for nuclear genome annotation.

Table S2. Statistics of gene models predicted under different BRAKER3 parameter settings for *Carya glabra* haplotype A.

Table S3. Genomic data used for phylogenomic analysis.

Table S4. Annotated genes in the *Carya glabra* chloroplast genome.

Table S5. Annotated genes in the *Carya glabra* mitchondrial genome.

Table S6. Chloroplast-derived segments in the *Carya glabra* mitochondrial genome.

Table S7. Omni-C library quality control report from Phase Genomics’ hic_qc pipeline.

Table S8. Lengths (in Mb) of the 64 assembled pseudo-chromosomes of *Carya glabra*.

Table S9. Summary of repetitive element annotation in *Carya glabra*.

Table S10. Statistics of finalized gene models predicted for four haplotypes from *Carya glabra*.

Table S11. Four major classes of plant disease resistance genes (*R* genes) identified in *Carya glabra* and three other *Carya* species with assembled genomes.

Table S12. Putative *Carya glabra* plant disease resistance genes (*R* genes) identified in the syntenic regions corresponding to the major quantitative trait locus (QTL) associated with phylloxera resistance in *Carya illinoinensis*.

Table S13. Misannotated and missing genes in previously published *Carya glabra* chloroplast genomes.

Table S14. Shared and unique mitochondrial genes among *Carya glabra*, *Carya illinoinensis*, and *Juglans mandshurica*.

Fig. S1. Dot plot comparing one set of chromosomes from *Carya illinoinensis* (2*x*) with the unitig assembly of *Carya glabra* (4*x*). The zoomed-in panel shows that each *C. illinoinensis* region (shaded in yellow) corresponds to four unitigs from *C. glabra* (shaded in green), a pattern observed across the entire *C. illinoinensis* genome.

Fig. S2. Manual curation of the YaHS scaffolding output using Juicebox. (A) The Hi-C contact map generated in Juicebox was used to identify scaffolding errors. Two examples, one misjoin and one inversion error, are shown. In the contact map, each blue square represents a scaffold, and the green squares within a scaffold represent unitigs anchored to that scaffold. Based on the Hi-C signals, the misjoined scaffold was split into two scaffolds, while for the scaffold with an inversion error, the inverted unitigs (yellow square) were selected and inverted. (B) Dot plots comparing the *Carya illinoinensis* genome with the *Carya glabra* scaffolds before and after manual curation are shown.

Fig. S3. Venn diagram showing shared and unique mitochondrial genes among *Carya glabra*, *Carya illinoinensis*, and *Juglans mandshurica*.

Table S1. Protein evidence used for nuclear genome annotation.

| Species | Source | Version/Accession |
| --- | --- | --- |
| *Arabidopsis thaliana* (Brassicaceae) | Phytozome | Araport11 |
| *Cucumis sativus* (Cucurbitaceae) | Phytozome | v1.0 |
| *Eucalyptus grandis* (Myrtaceae) | Phytozome | v2.0 |
| *Glycine max* (Fabaceae) | Phytozome | Wm82.a6.v1 |
| *Malus domestica* (Rosaceae) | Phytozome | v1.1 |
| *Populus trichocarpa* (Salicaceae) | Phytozome | v4.1 |
| *Prunus persica* (Rosaceae) | Phytozome | v2.1 |
| *Vitis vinifera* (Vitaceae) | Phytozome | v2.1 |
| *Carya cathayensis* (Juglandaceae) | Juglandaceae Genome Projects^1^ | v1.0 |
| *Carya illinoinensis* (Juglandaceae) | Phytozome | Pawnee v1.1 |
| *Carya sinensis* (Juglandaceae) | Juglandaceae Genome Projects | v1.0 |
| *Engelhardia roxburghiana* (Juglandaceae) | Juglandaceae Genome Projects | v1.0 |
| *Juglans regia* (Juglandaceae) | NCBI | GCA_001411555.2 |
| *Pterocarya stenoptera* (Juglandaceae) | Juglandaceae Genome Projects | v2.0 |

Note: ^1^<https://cmb.bnu.edu.cn/juglans/>

Table S2. Statistics of gene models predicted under different BRAKER3 parameter settings for *Carya glabra* haplotype A.

| Method | RNA-Seq data from *C. glabra* and protein data from nine model species^1^ | | |  | RNA-Seq data from *C. glabra* and protein data from OrthoDB^2^ (adding *C. illinoensis*) | | |  | RNA-Seq data from *C. glabra* and protein data from 11 model species^3^ | | |  | RNA-Seq data from *C. glabra* and protein data from 14 model species^4^ | | |
| --- | --- | --- | --- | --- | --- | --- | --- | --- | --- | --- | --- | --- | --- | --- | --- |
| Filter | None | 100 aa^5^ | 50 aa^6^ |  | None | 100 aa | 50 aa |  | None | 100 aa | 50 aa |  | None | 100 aa | 50 aa |
| Gene no. | 29,496 | 27,756 | 29,379 |  | 30,574 | 28,573 | 30,440 |  | 31,294 | 29,140 | 31,160 |  | 31,085 | 28,996 | 30,947 |
| Mean gene length (bp) | 4,558 | 4,787 | 4,574 |  | 4,444 | 4,698 | 4,461 |  | 4,357 | 4,620 | 4,374 |  | 4,380 | 4,639 | 4,398 |
| Mean CDS length (bp) | 1,268 | 1,333 | 1,273 |  | 1,248 | 1,319 | 1,253 |  | 1,229 | 1,302 | 1,234 |  | 1,236 | 1,308 | 1,241 |
| Average exons per gene | 5.2 | 5.4 | 5.2 |  | 5.0 | 5.3 | 5.0 |  | 5.0 | 5.2 | 5.0 |  | 5.0 | 5.2 | 5.0 |
| Mono-/multi-exonic ratio^7^ | 0.25 | 0.24 | 0.25 |  | 0.27 | 0.25 | 0.27 |  | 0.27 | 0.26 | 0.27 |  | 0.28 | 0.26 | 0.28 |
| BUSCO | 97.6% | 97.1% | 97.6% |  | 97.5% | 97.1% | 97.5% |  | 97.6% | 97.1% | 97.6% |  | 97.7% | 97.1% | 97.7% |

Note: the statistics are based on the longest isoform of each gene. ^1^the nine model species include *Arabidopsis thaliana*, *Cucumis sativus*, *Eucalyptus grandis*, *Glycine max*, *Malus domestica*, *Populus trichocarpa*, *Prunus persica*, *Vitis vinifera*, and *Carya illinoinensis*. ^2^Viridiplantae database from the OrthoDB v.12 was used. ^3^the 11 species include *Arabidopsis thaliana*, *Cucumis sativus*, *Eucalyptus grandis*, *Glycine max*, *Malus domestica*, *Populus trichocarpa*, *Prunus persica*, *Vitis vinifera*, *Carya cathayensis*, *Carya illinoinensis*, and *Carya sinensis*. ^4^the 14 model species include *Arabidopsis thaliana*, *Cucumis sativus*, *Eucalyptus grandis*, *Glycine max*, *Malus domestica*, *Populus trichocarpa*, *Prunus persica*, *Vitis vinifera*, *Carya cathayensis*, *Carya illinoinensis*, *Carya sinensis*, *Engelhardia roxburghiana*, *Juglans regia*, and *Pterocarya stenoptera*. ^5^gene models meeting any of the following criteria were filtered out: (1) presence of a premature stop codon; (2) absence of a start and/or stop codon; or (3) an open reading frame (ORF) length of ≤100 amino acids. ^6^gene models meeting any of the following criteria were filtered out: (1) presence of a premature stop codon; (2) absence of a start and/or stop codon; or (3) an ORF length of ≤50 amino acids. ^7^the ratio of mono-exnoic (single-exon) to multi-exonic (multiple-exon) genes.

Table S3. Genomic data used for phylogenomic analysis. SRA: Sequence Read Archive.

| Species | No. of samples | SRA accession number or genome assembly |
| --- | --- | --- |
| *Carya aquatica* | 4 | SRR13361464, SRR14065918, SRR26756448, SRR6804841 |
| *Carya cathayensis* | 1 | Genome assembly (v1.0) from Juglandaceae Genome Projects^1^ |
| *Carya cordiformis* | 3 | SRR13370650, SRR26756242, SRR6804840 |
| *Carya dabieshanensis* | 2 | SRR20492969, SRR6804849 |
| *Carya floridana* | 1 | SRR6804844 |
| *Carya glabra* | 1 | Genome assembly (haplotype A) from the current study |
| *Carya hunanensis* | 1 | Genome assembly (v1.0; haplotype E) from Juglandaceae Genome Projects |
| *Carya illinoinensis* | 1 | Genome assembly (Pawnee v1.1) from Phytozome |
| *Carya kweichowensis* | 2 | SRR20493000, SRR6804852 |
| *Carya laciniosa* | 1 | SRR6804855 |
| *Carya myristiciformis* | 1 | SRR6804845 |
| *Carya ovata* | 3 | SRR13300627, SRR6804843, SRR29124870 |
| *Carya palmeri* | 1 | SRR6804847 |
| *Carya poilanei* | 1 | SRR17248067 |
| *Carya sinensis* | 1 | Genome assembly (v1.0) from Juglandaceae Genome Projects |
| *Carya texana* | 1 | SRR6804850 |
| *Carya tomentosa* | 1 | SRR6804842 |
| *Carya tonkinensis* | 2 | SRR17248066, SRR6804856 |
| *Juglans regia* | 1 | Genome assembly (Walnut 2.0) from Ensembl Plants |

Note: ^1^<https://cmb.bnu.edu.cn/juglans/>

Table S4. Annotated genes in the *Carya glabra* chloroplast genome. LSC: large single copy; SSC: small single copy; IR: inverted repeat.

| No. | Gene ID | Gene product | Type | Location |
| --- | --- | --- | --- | --- |
| 1 | *accD* | acetyl-CoA carboxylase beta subunit | Protein coding | LSC |
| 2 | *atpA* | ATP synthase CF1 alpha subunit | Protein coding | LSC |
| 3 | *atpB* | ATP synthase CF1 beta subunit | Protein coding | LSC |
| 4 | *atpE* | ATP synthase CF1 epsilon subunit | Protein coding | LSC |
| 5 | *atpF* | ATP synthase CF0 B subunit | Protein coding | LSC |
| 6 | *atpH* | ATP synthase CF0 C subunit | Protein coding | LSC |
| 7 | *atpI* | ATP sccsynthase CF0 A subunit | Protein coding | LSC |
| 8 | *ccsA* | cytochrome c biogenesis protein | Protein coding | SSC |
| 9 | *cemA* | envelope membrane protein | Protein coding | LSC |
| 10 | *clpP1* | ATP-dependent Clp protease proteolytic subunit 1 | Protein coding | LSC |
| 11 | *infA* | translation initiation factor IF-1 | Protein coding | LSC |
| 12 | *matK* | maturase K | Protein coding | LSC |
| 13 | *ndhA* | NADH dehydrogenase subunit 1 | Protein coding | SSC |
| 14 | *ndhB* | NADH dehydrogenase subunit 2 | Protein coding | IR |
| 15 | *ndhC* | NADH dehydrogenase subunit 3 | Protein coding | LSC |
| 16 | *ndhD* | NADH dehydrogenase subunit 4 | Protein coding | SSC |
| 17 | *ndhE* | NADH dehydrogenase subunit 4L | Protein coding | SSC |
| 18 | *ndhF* | NADH dehydrogenase subunit 5 | Protein coding | SSC |
| 19 | *ndhG* | NADH dehydrogenase subunit 6 | Protein coding | SSC |
| 20 | *ndhH* | NADH dehydrogenase subunit 7 | Protein coding | SSC |
| 21 | *ndhI* | NADH dehydrogenase subunit I | Protein coding | SSC |
| 22 | *ndhJ* | NADH dehydrogenase subunit J | Protein coding | LSC |
| 23 | *ndhK* | NADH dehydrogenase subunit K | Protein coding | LSC |
| 24 | *pafI* | photosystem I assembly protein Ycf3 | Protein coding | LSC |
| 25 | *pafII* | photosystem I assembly protein Ycf4 | Protein coding | LSC |
| 26 | *pbf1* | photosystem biogenesis factor 1 | Protein coding | LSC |
| 27 | *petA* | cytochrome f | Protein coding | LSC |
| 28 | *petB* | cytochrome b6 | Protein coding | LSC |
| 29 | *petD* | cytochrome b6/f complex subunit IV | Protein coding | LSC |
| 30 | *petG* | cytochrome b6/f complex subunit V | Protein coding | LSC |
| 31 | *petL* | cytochrome b6/f complex subunit VI | Protein coding | LSC |
| 32 | *petN* | cytochrome b6/f complex subunit N | Protein coding | LSC |
| 33 | *psaA* | photosystem I P700 chlorophyll a apoprotein A1 | Protein coding | LSC |
| 34 | *psaB* | photosystem I P700 chlorophyll a apoprotein A2 | Protein coding | LSC |
| 35 | *psaC* | photosystem I iron-sulfur center | Protein coding | SSC |
| 36 | *psaI* | photosystem I reaction center subunit VIII | Protein coding | LSC |
| 37 | *psaJ* | photosystem I reaction center subunit IX | Protein coding | LSC |
| 38 | *psbA* | photosystem II protein D1 | Protein coding | LSC |
| 39 | *psbB* | photosystem II 47 kDa protein | Protein coding | LSC |
| 40 | *psbC* | photosystem II 44 kDa protein | Protein coding | LSC |
| 41 | *psbD* | photosystem II protein D2 | Protein coding | LSC |
| 42 | *psbE* | photosystem II protein V | Protein coding | LSC |
| 43 | *psbF* | photosystem II protein VI | Protein coding | LSC |
| 44 | *psbH* | photosystem II protein H | Protein coding | LSC |
| 45 | *psbI* | photosystem II protein I | Protein coding | LSC |
| 46 | *psbJ* | photosystem II protein J | Protein coding | LSC |
| 47 | *psbK* | photosystem II protein K | Protein coding | LSC |
| 48 | *psbL* | photosystem II protein L | Protein coding | LSC |
| 49 | *psbM* | photosystem II protein M | Protein coding | LSC |
| 50 | *psbT* | photosystem II protein T | Protein coding | LSC |
| 51 | *psbZ* | photosystem II protein Z | Protein coding | LSC |
| 52 | *rbcL* | ribulose-1,5-bisphosphate carboxylase/oxygenase large subunit | Protein coding | LSC |

Table S4 Continued

| No. | Gene ID | Gene product | Type | Location |
| --- | --- | --- | --- | --- |
| 53 | *rpl14* | ribosomal protein L14 | Protein coding | LSC |
| 54 | *rpl16* | ribosomal protein L16 | Protein coding | LSC |
| 55 | *rpl2* | ribosomal protein L2 | Protein coding | IR |
| 56 | *rpl20* | ribosomal protein L20 | Protein coding | LSC |
| 57 | *rpl22* | ribosomal protein L22 | Protein coding | LSC |
| 58 | *rpl23* | ribosomal protein L23 | Protein coding | IR |
| 59 | *rpl32* | ribosomal protein L32 | Protein coding | SSC |
| 60 | *rpl33* | ribosomal protein L33 | Protein coding | LSC |
| 61 | *rpl36* | ribosomal protein L36 | Protein coding | LSC |
| 62 | *rpoA* | RNA polymerase alpha subunit | Protein coding | LSC |
| 63 | *rpoB* | RNA polymerase beta subunit | Protein coding | LSC |
| 64 | *rpoC1* | RNA polymerase beta' subunit | Protein coding | LSC |
| 65 | *rpoC2* | RNA polymerase beta'' subunit | Protein coding | LSC |
| 66 | *rps11* | ribosomal protein S11 | Protein coding | LSC |
| 67 | *rps12* | ribosomal protein S12 | Protein coding | IR |
| 68 | *rps14* | ribosomal protein S14 | Protein coding | LSC |
| 69 | *rps15* | ribosomal protein S15 | Protein coding | SSC |
| 70 | *rps16* | ribosomal protein S16 | Protein coding | LSC |
| 71 | *rps18* | ribosomal protein S18 | Protein coding | LSC |
| 72 | *rps19* | ribosomal protein S19 | Protein coding | LSC |
| 73 | *rps2* | ribosomal protein S2 | Protein coding | LSC |
| 74 | *rps3* | ribosomal protein S3 | Protein coding | LSC |
| 75 | *rps4* | ribosomal protein S4 | Protein coding | LSC |
| 76 | *rps7* | ribosomal protein S7 | Protein coding | IR |
| 77 | *rps8* | ribosomal protein S8 | Protein coding | LSC |
| 78 | *ycf1* | Ycf1 protein | Protein coding | SSC, IR |
| 79 | *ycf2* | Ycf2 protein | Protein coding | IR |
| 80 | *trnA-UGC* | tRNA-Ala | tRNA | IR |
| 81 | *trnC-GCA* | tRNA-Cys | tRNA | LSC |
| 82 | *trnD-GUC* | tRNA-Asp | tRNA | LSC |
| 83 | *trnE-UUC* | tRNA-Glu | tRNA | LSC |
| 84 | *trnF-GAA* | tRNA-Phe | tRNA | LSC |
| 85 | *trnfM-CAU* | tRNA-fMet | tRNA | LSC |
| 86 | *trnG-GCC* | tRNA-Gly | tRNA | LSC |
| 87 | *trnG-UCC* | tRNA-Gly | tRNA | LSC |
| 88 | *trnH-GUG* | tRNA-His | tRNA | LSC |
| 89 | *trnI-CAU* | tRNA-Ile | tRNA | IR |
| 90 | *trnI-GAU* | tRNA-Ile | tRNA | IR |
| 91 | *trnK-UUU* | tRNA-Lys | tRNA | LSC |
| 92 | *trnL-CAA* | tRNA-Leu | tRNA | IR |
| 93 | *trnL-UAA* | tRNA-Leu | tRNA | LSC |
| 94 | *trnL-UAG* | tRNA-Leu | tRNA | SSC |
| 95 | *trnM-CAU* | tRNA-Met | tRNA | LSC |
| 96 | *trnN-GUU* | tRNA-Asn | tRNA | IR |
| 97 | *trnP-UGG* | tRNA-Pro | tRNA | LSC |
| 98 | *trnQ-UUG* | tRNA-Gln | tRNA | LSC |
| 99 | *trnR-ACG* | tRNA-Arg | tRNA | IR |
| 100 | *trnR-UCU* | tRNA-Arg | tRNA | LSC |
| 101 | *trnS-GCU* | tRNA-Ser | tRNA | LSC |
| 102 | *trnS-GGA* | tRNA-Ser | tRNA | LSC |
| 103 | *trnS-UGA* | tRNA-Ser | tRNA | LSC |
| 104 | *trnT-GGU* | tRNA-Thr | tRNA | LSC |
| 105 | *trnT-UGU* | tRNA-Thr | tRNA | LSC |

Table S4 Continued

| No. | Gene ID | Gene product | Type | Location |
| --- | --- | --- | --- | --- |
| 106 | *trnV-GAC* | tRNA-Val | tRNA | IR |
| 107 | *trnV-UAC* | tRNA-Val | tRNA | LSC |
| 108 | *trnW-CCA* | tRNA-Trp | tRNA | LSC |
| 109 | *trnY-GUA* | tRNA-Tyr | tRNA | LSC |
| 110 | *rrn16* | 16S ribosomal RNA | rRNA | IR |
| 111 | *rrn23* | 23S ribosomal RNA | rRNA | IR |
| 112 | *rrn4.5* | 4.5S ribosomal RNA | rRNA | IR |
| 113 | *rrn5* | 5S ribosomal RNA | rRNA | IR |

Table S5. Annotated genes in the *Carya glabra* mitochondrial genome. The mitogenome includes two chromosomes: mtChr1 and mtChr2.

| No. | Gene | Gene product | Type | Location | Origin |
| --- | --- | --- | --- | --- | --- |
| 1 | *atp1* | ATPase subunit 1 | Protein coding | mtChr1 | Native |
| 2 | *atp4* | ATPase subunit 4 | Protein coding | mtChr1 | Native |
| 3 | *atp6* | ATPase subunit 6 | Protein coding | mtChr1 | Native |
| 4 | *atp8* | ATPase subunit 8 | Protein coding | mtChr1 | Native |
| 5 | *atp9* | ATPase subunit 9 | Protein coding | mtChr1, mtChr2 | Native |
| 6 | *ccmB* | cytochrome c biogenesis B | Protein coding | mtChr1, mtChr2 | Native |
| 7 | *ccmC* | cytochrome c biogenesis C | Protein coding | mtChr1 | Native |
| 8 | *ccmFC* | cytochrome c biogenesis FC | Protein coding | mtChr1 | Native |
| 9 | *ccmFN* | cytochrome b biogenesis FN | Protein coding | mtChr1, mtChr2 | Native |
| 10 | *cob* | apocytochrome b | Protein coding | mtChr1 | Native |
| 11 | *cox1* | cytochrome c oxidase subunit 1 | Protein coding | mtChr1 | Native |
| 12 | *cox2* | cytochrome c oxidase subunit 2 | Protein coding | mtChr1, mtChr2 | Native |
| 13 | *cox3* | cytochrome c oxidase subunit 3 | Protein coding | mtChr1 | Native |
| 14 | *matR* | maturase | Protein coding | mtChr1, mtChr2 | Native |
| 15 | *mttB* | transport membrane protein | Protein coding | mtChr1 | Native |
| 16 | *nad1* | NADH dehydrogenase subunit 1 | Protein coding | mtChr1, mtChr2 | Native |
| 17 | *nad2* | NADH dehydrogenase subunit 2 | Protein coding | mtChr1, mtChr2 | Native |
| 18 | *nad3* | NADH dehydrogenase subunit 3 | Protein coding | mtChr1, mtChr2 | Native |
| 19 | *nad4* | NADH dehydrogenase subunit 4 | Protein coding | mtChr1 | Native |
| 20 | *nad4L* | NADH dehydrogenase subunit 4L | Protein coding | mtChr1 | Native |
| 21 | *nad5* | NADH dehydrogenase subunit 5 | Protein coding | mtChr1, mtChr2 | Native |
| 22 | *nad6* | NADH dehydrogenase subunit 6 | Protein coding | mtChr1 | Native |
| 23 | *nad7* | NADH dehydrogenase subunit 7 | Protein coding | mtChr1 | Native |
| 24 | *nad9* | NADH dehydrogenase subunit 9 | Protein coding | mtChr1 | Native |
| 25 | *rpl10* | ribosomal protein L10 | Protein coding | mtChr1, mtChr2 | Native |
| 26 | *rpl16* | ribosomal protein L16 | Protein coding | mtChr1 | Native |
| 27 | *rpl2* | ribosomal protein L2 | Protein coding | mtChr1, mtChr2 | Native |
| 28 | *rpl5* | ribosomal protein L5 | Protein coding | mtChr1 | Native |
| 29 | *rps1* | ribosomal protein S1 | Protein coding | mtChr1 | Native |
| 30 | *rps10* | ribosomal protein S10 | Protein coding | mtChr1, mtChr2 | Native |
| 31 | *rps12* | ribosomal protein S12 | Protein coding | mtChr1, mtChr2 | Native |
| 32 | *rps14* | ribosomal protein S14 | Protein coding | mtChr1 | Native |
| 33 | *rps19* | ribosomal protein S19 | Protein coding | mtChr1 | Native |
| 34 | *rps3* | ribosomal protein S3 | Protein coding | mtChr1 | Native |
| 35 | *rps4* | ribosomal protein S4 | Protein coding | mtChr1 | Native |
| 36 | *sdh3* | succinate dehydrogenase subunit 3 | Protein coding | mtChr1, mtChr2 | Native |
| 37 | *sdh4* | succinate dehydrogenase subunit 4 | Protein coding | mtChr1 | Native |
| 38 | *petN* | cytochrome b6/f complex subunit N | Protein coding | mtChr1 | Plastome |
| 39 | *psaJ* | photosystem I reaction center subunit IX | Protein coding | mtChr1 | Plastome |
| 40 | *psbJ* | photosystem II protein J | Protein coding | mtChr1 | Plastome |
| 41 | *rpl20* | ribosomal protein L20 | Protein coding | mtChr1 | Plastome |
| 42 | *rpl33* | ribosomal protein L33 | Protein coding | mtChr1 | Plastome |
| 43 | *rrn18* | 18S ribosomal RNA | rRNA | mtChr1 | Native |
| 44 | *rrn26* | 26S ribosomal RNA | rRNA | mtChr1 | Native |
| 45 | *rrn5* | 5S ribosomal RNA | rRNA | mtChr1 | Native |
| 46 | *trnC-GCA* | tRNA-Cys | tRNA | mtChr1 | Bacteria |
| 47 | *trnC-GCA* | tRNA-Cys | tRNA | mtChr1 | Native |
| 48 | *trnE-UUC* | tRNA-Glu | tRNA | mtChr1 | Native |
| 49 | *trnF-GAA* | tRNA-Phe | tRNA | mtChr1 | Native |
| 50 | *trnfM-CAU* | tRNA-Met | tRNA | mtChr1 | Native |
| 51 | *trnG-GCC* | tRNA-Gly | tRNA | mtChr1 | Native |
| 52 | *trnI-CAU* | tRNA-Ile | tRNA | mtChr1 | Native |

Table S5 Continued

| No. | Gene | Gene product | Type | Location | Origin |
| --- | --- | --- | --- | --- | --- |
| 53 | *trnK-UUU* | tRNA-Lys | tRNA | mtChr1, mtChr2 | Native |
| 54 | *trnP-UGG* | tRNA-Pro | tRNA | mtChr1 | Native |
| 55 | *trnQ-UUG* | tRNA-Gln | tRNA | mtChr1 | Native |
| 56 | *trnS-GCU* | tRNA-Ser | tRNA | mtChr1 | Native |
| 57 | *trnS-UGA* | tRNA-Ser | tRNA | mtChr1 | Native |
| 58 | *trnY-GUA* | tRNA-Tyr | tRNA | mtChr1 | Native |
| 59 | *trnA-UGC* | tRNA-Ala | tRNA | mtChr1 | Plastome |
| 60 | *trnD-GUC* | tRNA-Asp | tRNA | mtChr1 | Plastome |
| 61 | *trnH-GUG* | tRNA-His | tRNA | mtChr1 | Plastome |
| 62 | *trnI-CAU* | tRNA-Ile | tRNA | mtChr1 | Plastome |
| 63 | *trnL-CAA* | tRNA-Leu | tRNA | mtChr1 | Plastome |
| 64 | *trnM-CAU* | tRNA-Met | tRNA | mtChr1 | Plastome |
| 65 | *trnN-GUU* | tRNA-Asn | tRNA | mtChr1 | Plastome |
| 66 | *trnP-UGG* | tRNA-Pro | tRNA | mtChr1 | Plastome |
| 67 | *trnV-GAC* | tRNA-Val | tRNA | mtChr1 | Plastome |
| 68 | *trnW-CCA* | tRNA-Trp | tRNA | mtChr1, mtChr2 | Plastome |

Table S6. Chloroplast-derived segments in the *Carya glabra* mitochondrial genome. ^*^Fragmentary gene; ^+^gene containing premature stop codon.

| Location | Aligned length (bp) | Identity to plastome (%) | Plastome gene(s) |
| --- | --- | --- | --- |
| mtChr1: 335851-350810 | 15,031 | 99.2 | *trnA-UGC*, *trnI-CAU*, *trnL-CAA*, *trnV-GAC*, *rps12*^*^, *rpl23*^*^, *rrn16*^*^, *ndhB*^+^, *rps7*^+^, *ycf2*^+^ |
| mtChr1: 366061-367918 | 2,137 | 84.3 | *psaJ*, *rpl20*, *rpl33*, *rps18*^+^ |
| mtChr1: 288398-289764 | 1,379 | 99.0 | *rpl2*^*^, *rpl23*^*^ |
| mtChr1: 71318-72550 | 1,233 | 71.4 | *trnP-UGG*, *trnW-CCA*, *petG*^+^, *petL*^+^ |
| mtChr1: 441717-442798 | 1,083 | 94.7 | *trnD-GUC* |
| mtChr1: 367903-368823 | 936 | 97.1 | *petN* |
| mtChr1: 102562-103322 | 769 | 92.9 | *psaA*^*^, *psaB*^*^ |
| mtChr1: 72510-73190 | 681 | 99.7 | *rpoC2*^*^ |
| mtChr1: 249552-249976 | 425 | 99.5 | *trnI-GAU*^*^ |
| mtChr1: 357130-357276 | 147 | 90.5 | *psbJ* |
| mtChr1: 283627-283714 | 88 | 95.5 | *trnH-GUG* |
| mtChr1: 474776-474858 | 83 | 100.0 | *trnN-GUU* |
| mtChr1: 153840-153917 | 78 | 94.9 | *trnM-CAU* |
| mtChr1: 376921-376997 | 77 | 84.4 | *trnM-CAU* |

Table S7. Omni-C library quality control report from Phase Genomics’ hic_qc pipeline.

| Category | Omni-C library statistics | Expected values |
| --- | --- | --- |
| Percentage of read pairs spanning at least 10 kb among all high-quality read pairs mapped to the same unitig of at least 10 kb in length | 6.7% | >3.0% |
| Percentage of high-quality read pairs mapping to two different unitigs, each greater than 10 kb in length | 7.1% | >2.5% |

Note: high-quality read pairs are defined as those with a minimum mapping quality of 20, maximum edit distance of 5, and no duplicate reads.

Table S8. Lengths (in Mb) of the 64 assembled pseudo-chromosomes of *Carya glabra*.

| Pseudo-chromosome | Haplotype A | Haplotype B | Haplotype C | Haplotype D |
| --- | --- | --- | --- | --- |
| 1 | 53.0 | 52.6 | 52.1 | 49.6 |
| 2 | 36.2 | 35.2 | 35.2 | 34.3 |
| 3 | 50.2 | 48.6 | 47.3 | 47.2 |
| 4 | 37.5 | 36.7 | 36.2 | 36.2 |
| 5 | 45.4 | 43.7 | 43.0 | 42.0 |
| 6 | 31.5 | 30.6 | 30.6 | 30.3 |
| 7 | 39.6 | 39.4 | 37.8 | 36.9 |
| 8 | 33.6 | 32.9 | 32.0 | 31.1 |
| 9 | 42.3 | 40.5 | 39.9 | 39.8 |
| 10 | 33.8 | 33.5 | 33.1 | 32.9 |
| 11 | 41.2 | 41.1 | 39.6 | 38.8 |
| 12 | 24.9 | 24.7 | 23.4 | 20.9 |
| 13 | 32.0 | 31.9 | 31.6 | 28.8 |
| 14 | 30.5 | 29.4 | 29.2 | 28.5 |
| 15 | 41.4 | 37.7 | 36.8 | 35.4 |
| 16 | 27.5 | 26.9 | 26.8 | 26.7 |

Table S9. Summary of repetitive element annotation in *Carya glabra*.

| Category |  | Hap. A | Hap. B | Hap. C | Hap. D |
| --- | --- | --- | --- | --- | --- |
| Transposable elements | Class I: retrotransposons | 27.2% | 27.1% | 24.7% | 26.9% |
|  | Class II: DNA transposons | 19.5% | 19.9% | 21.5% | 19.4% |
| Simple repeats |  | 1.2% | 1.2% | 1.3% | 1.3% |
| Low complexity |  | 0.2% | 0.2% | 0.2% | 0.2% |
| Unclassified interspersed repeats |  | 6.9% | 6.1% | 6.2% | 6.0% |
|  |  |  |  |  |  |
| Total |  | 55.0% | 54.4% | 54.0% | 53.8% |

Table S10. Statistics of finalized gene models predicted for four haplotypes from *Carya glabra*.

| Haplotype | A | B | C | D |
| --- | --- | --- | --- | --- |
| Gene no. | 30,947 | 31,087 | 30,369 | 30,110 |
| Average gene length (bp) | 4,398 | 4,364 | 4,460 | 4,381 |
| Average CDS length (bp) | 1,241 | 1,239 | 1,254 | 1,240 |
| Average exons per gene | 5.0 | 5.0 | 5.0 | 5.0 |
| BUSCO (%) | 97.7 | 97.1 | 96.5 | 94.9 |
| Genes assigned to gene families (%) | 94.3 | 93.9 | 94.7 | 94.4 |
| Genes annotated with GO terms (%) | 79.7 | 79.4 | 79.9 | 79.4 |
| Genes annotated with protein domains (%) | 86.3 | 85.9 | 86.5 | 86.3 |
| Core gene family completeness | 0.982 | 0.982 | 0.978 | 0.967 |

Note: the statistics are based on the longest isoform of each gene. GO: gene ontology.

Table S11. Four major classes of plant disease resistance genes (*R* genes) identified in *Carya glabra* and three other *Carya* species with assembled genomes.

| *R* gene class | *C. glabra* | | | | *C. illinoinensis*^1^ | *C. sinensis*^2^ | *C. cathayensis*^2^ |
| --- | --- | --- | --- | --- | --- | --- | --- |
|  | Hap. A | Hap. B | Hap. C | Hap. D |  |  |  |
| CNL | 56 | 61 | 59 | 41 | 70 | 44 | 29 |
| TNL | 39 | 83 | 96 | 34 | 65 | 11 | 26 |
| RLP | 214 | 192 | 189 | 226 | 217 | 300 | 375 |
| RLK | 316 | 302 | 311 | 307 | 372 | 330 | 370 |
| Total | 625 | 638 | 655 | 608 | 724 | 685 | 800 |

Note: the CNL protein contains the coiled-coil domain, the nucleotide-binding site (NBS) domain, and the leucine-rich repeat (LRR) domain. The TNL protein contains the Toll-interleukin receptor-like domain, the NBS domain, and the LRR domain. The RLP (receptor-like protein) protein contains the transmembrane (TM) domain and the LRR domain. The RLK (receptor-like kinase) protein contains the TM domain, the LRR domain, and the kinase domain. ^1^The statistics are from *C. illinoinensis* cv. ‘Pawnee’ (Lovell et al. 2021). ^2^Both genomes are from Zhang et al. 2024b. Hap.: haplotype.

Table S12. Putative *Carya glabra* plant disease resistance genes (*R* genes) identified in the syntenic regions corresponding to the major quantitative trait locus (QTL) associated with phylloxera resistance in *Carya illinoinensis*.

| Haplotype | Gene name | *R* gene class | Location | Syntenic gene in  *C. illinoinensis* | % identity |
| --- | --- | --- | --- | --- | --- |
| A | dhCarGlab.v1a1.Chr16A.g01360.t1 | TNL | Chr. 16A: 1973676 - 1977343 |  |  |
| A | dhCarGlab.v1a1.Chr16A.g01410.t1 | TNL | Chr. 16A: 2185240 - 2190125 |  |  |
| A | dhCarGlab.v1a1.Chr16A.g01420.t1 | RLP | Chr. 16A: 2268899 - 2269806 |  |  |
| A | dhCarGlab.v1a1.Chr16A.g01430.t1 | TNL | Chr. 16A: 2270798 - 2275685 |  |  |
| A | dhCarGlab.v1a1.Chr16A.g01450.t1 | TNL | Chr. 16A: 2291459 - 2295800 |  |  |
| A | dhCarGlab.v1a1.Chr16A.g01460.t1 | TNL | Chr. 16A: 2325172 - 2331391 |  |  |
| A | dhCarGlab.v1a1.Chr16A.g01480.t1 | TNL | Chr. 16A: 2412331 - 2417424 |  |  |
| A | dhCarGlab.v1a1.Chr16A.g01500.t1 | RLK | Chr. 16A: 2433195 - 2436628 |  |  |
| B | dhCarGlab.v1a1.Chr16B.g01550.t1 | TNL | Chr. 16B: 2162361 - 2165884 |  |  |
| B | dhCarGlab.v1a1.Chr16B.g01560.t2 | TNL | Chr. 16B: 2199479 - 2204805 |  |  |
| B | dhCarGlab.v1a1.Chr16B.g01570.t1 | TNL | Chr. 16B: 2271505 - 2275301 |  |  |
| B | dhCarGlab.v1a1.Chr16B.g01600.t2 | TNL | Chr. 16B: 2291370 - 2296652 |  |  |
| B | dhCarGlab.v1a1.Chr16B.g01630.t2 | TNL | Chr. 16B: 2357117 - 2363523 |  |  |
| B | dhCarGlab.v1a1.Chr16B.g01640.t2 | RLP | Chr. 16B: 2370716 - 2371754 |  |  |
| B | dhCarGlab.v1a1.Chr16B.g01650.t1 | TNL | Chr. 16B: 2371918 - 2375633 |  |  |
| B | dhCarGlab.v1a1.Chr16B.g01680.t1 | TNL | Chr. 16B: 2444556 - 2449470 |  |  |
| B | dhCarGlab.v1a1.Chr16B.g01700.t1 | TNL | Chr. 16B: 2468763 - 2473767 |  |  |
| B | dhCarGlab.v1a1.Chr16B.g01770.t1 | RLK | Chr. 16B: 2535654 - 2539083 |  |  |
| C | dhCarGlab.v1a1.Chr16C.g01400.t1 | TNL | Chr. 16C: 2136794 - 2140456 | CiLak.16G014000.1 | 70.4 |
| C | dhCarGlab.v1a1.Chr16C.g01410.t2 | TNL | Chr. 16C: 2153782 - 2158982 | CiLak.16G014100.1 | 66.0 |
| C | dhCarGlab.v1a1.Chr16C.g01440.t1 | TNL | Chr. 16C: 2386033 - 2391055 | CiLak.16G014300.1 | 86.0 |
| C | dhCarGlab.v1a1.Chr16C.g01480.t1 | TNL | Chr. 16C: 2497010 - 2502078 | CiLak.16G014300.1 | 89.8 |
| C | dhCarGlab.v1a1.Chr16C.g01510.t1 | TNL | Chr. 16C: 2547531 - 2553573 | CiLak.16G014000.1 | 70.8 |
| C | dhCarGlab.v1a1.Chr16C.g01530.t1 | TNL | Chr. 16C: 2660559 - 2665364 | CiLak.16G014000.1 | 96.5 |
| C | dhCarGlab.v1a1.Chr16C.g01550.t1 | TNL | Chr. 16C: 2738972 - 2742700 | CiLak.16G013900.1 | 99.0 |
| C | dhCarGlab.v1a1.Chr16C.g01560.t1 | TNL | Chr. 16C: 2743843 - 2748369 | CiLak.16G014000.1 | 98.5 |
| C | dhCarGlab.v1a1.Chr16C.g01600.t1 | TNL | Chr. 16C: 2835169 - 2841464 | CiLak.16G014000.1 | 71.0 |
| C | dhCarGlab.v1a1.Chr16C.g01680.t1 | TNL | Chr. 16C: 2982302 - 2988728 | CiLak.16G014300.1 | 91.5 |
| C | dhCarGlab.v1a1.Chr16C.g01700.t1 | RLK | Chr. 16C: 3025435 - 3028868 | CiLak.16G014900.1 | 99.3 |

Table S12 Continued

| Haplotype | Gene name | *R* gene class | Location | Syntenic gene in  *C. illinoinensis* | % identity |
| --- | --- | --- | --- | --- | --- |
| D | dhCarGlab.v1a1.Chr16D.g01310.t1 | TNL | Chr. 16D: 1961595 - 1966668 |  |  |
| D | dhCarGlab.v1a1.Chr16D.g01320.t1 | TNL | Chr. 16D: 1986524 - 1991236 |  |  |
| D | dhCarGlab.v1a1.Chr16D.g01390.t1 | TNL | Chr. 16D: 2323753 - 2330174 |  |  |
| D | dhCarGlab.v1a1.Chr16D.g01420.t1 | RLP | Chr. 16D: 2521824 - 2522679 |  |  |
| D | dhCarGlab.v1a1.Chr16D.g01430.t1 | TNL | Chr. 16D: 2523457 - 2528122 |  |  |
| D | dhCarGlab.v1a1.Chr16D.g01440.t1 | TNL | Chr. 16D: 2545456 - 2549333 |  |  |
| D | dhCarGlab.v1a1.Chr16D.g01490.t1 | TNL | Chr. 16D: 2650932 - 2655992 |  |  |
| D | dhCarGlab.v1a1.Chr16D.g01520.t1 | RLK | Chr. 16D: 2694631 - 2698064 |  |  |

Note: the TNL protein contains the Toll-interleukin receptor-like domain, the nucleotide-binding site (NBS) domain, and the leucine-rich repeat (LRR) domain. The RLP (receptor-like protein) protein contains the transmembrane (TM) domain and the LRR domain. The RLK (receptor-like kinase) protein contains the TM domain, the LRR domain, and the kinase domain. Chr.: chromosome.

Table S13. Misannotated and missing genes in previously published *Carya glabra* chloroplast genomes.

| Gene ID | Gene product | Published *Carya glabra* chloroplast genomes on GenBank | | |
| --- | --- | --- | --- | --- |
|  |  | BK061156  (Luo et al. 2021) | OR099205  (Liu et al. 2025) | NC_067504  (Xi et al. 2022) |
| *atpB* | ATP synthase CF1 beta subunit |  | Missing |  |
| *infA* | translation initiation factor IF-1 |  |  | Missing |
| *petB* | cytochrome b6 |  |  | Exon 1 missing |
| *petD* | cytochrome b6/f complex subunit IV |  |  | Exon 1 missing |
| *psbB* | photosystem II 47 kDa protein |  | Misannotated as *psi* |  |
| *psbZ* | photosystem II protein Z |  | Misannotated as *lhbA* |  |
| *rpl16* | ribosomal protein L16 |  |  | Exon 1 missing |
| *rpoB* | RNA polymerase beta subunit |  | Missing |  |
| *ycf15* | putative chloroplast RF15 | Included | Included | Included |
| *trnA-UGC* | tRNA-Ala | Two additional copies misannotated at 105,498–105,557 and 144,984–145,043 |  | Two additional copies misannotated at 105,495–105,554 and 144,981–145,040 |
| *trnK-UUU* | tRNA-Lys | One additional copy misannotated at 6,403–6,462 |  | Misannotated at  6,400–6,459 |
| *tnrM-CAU* | tRNA-Met | Two additional copies misannotated at 91,947–92,021 and 158,520–158,594 | One additional copy misannotated at  35,273–35,331 |  |
| *trnP-GGG* | tRNA-Pro |  | Misannotated at  72,042–72,112 |  |
| *trnT-GGU* | tRNA-Thr |  | One additional copy misannotated at  57,114–57,172 |  |
| *rrn16* | 16S ribosomal RNA |  | Missing |  |
| *rrn23* | 23S ribosomal RNA |  | Missing |  |
| *rrn4.5* | 4.5S ribosomal RNA |  | Missing |  |
| *rrn5* | 5S ribosomal RNA |  | Missing |  |

Table S14. Shared and unique mitochondrial genes among *Carya glabra*, *Carya illinoinensis*, and *Juglans mandshurica*. ^*^Genes of plastome origin in *C. glabra*.

| Category | Gene number | Gene IDs |
| --- | --- | --- |
| Shared among all three species | 54 | *atp1*, *atp4*, *atp6*, *atp8*, *atp9*, *ccmB*, *ccmC*, *ccmFC*, *ccmFN*, *cob*, *cox1*, *cox2*, *cox3*, *matR*, *mttB*, *nad1*, *nad3*, *nad4*, *nad4L*, *nad5*, *nad6*, *nad7*, *nad9*, *rpl10*, *rpl16*, *rpl2*, *rpl5*, *rps1*, *rps10*, *rps12*, *rps14*, *rps19*, *rps4*, *rrn26*, *rrn5*, *sdh3*, *sdh4*, *trnC-GCA*, *trnD-GUC*, *trnE-UUC*, *trnF-GAA*, *trnG-GCC*, *trnH-GUG*, *trnK-UUU*, *trnL-CAA*, *trnM-CAU*, *trnN-GUU*, *trnP-UGG*, *trnQ-UUG*, *trnS-GCU*, *trnS-UGA*, *trnV-GAC*, *trnW-CCA*, *trnY-GUA* |
| Unique to *C. glabra* and *C. illinoinensis* | 2 | *nad2*, *rrn18* |
| Unique to *C. glabra* and *J. mandshurica* | 2 | *rps3*, *trnI-CAU* |
| Unique to *C. illinoinensis* and *J. mandshurica* | 1 | *rps7* |
| Unique to *C. glabra* | 7 | *petN*^*^, *psaJ*^*^, *psbJ*^*^, *rpl20*^*^, *rpl33*^*^, *trnA-UGC*^*^, *trnfM-CAU* |
| Unique to *J. mandshurica* | 3 | *rns*, *trnL-AAC*, *trnR-ACG* |
|  |  |  |
| Total | 69 |  |
